# Supplementary material for: Environmental data provide marginal benefit for predicting climate adaptation
Source: PLoS Genet. 2025 Jun 9;21(6):e1011714. doi: 10.1371/journal.pgen.1011714 (PMC12173371; doi:10.1371/journal.pgen.1011714)
Supplement: S1 File — All transfer plot model curves, where model is unconstrained to be negative. Panels represent fitness curves for individual trials, where individual points represent scaled BLUPs of an accession grown in that trial, plotted against an environmental variable (mean temperature, annual precipitation, elevation of origin). Each page contains panels for all trials where a phenotypic trait was measured, against a single environmental variable. (PDF) [file pgen.1011714.s007.pdf]

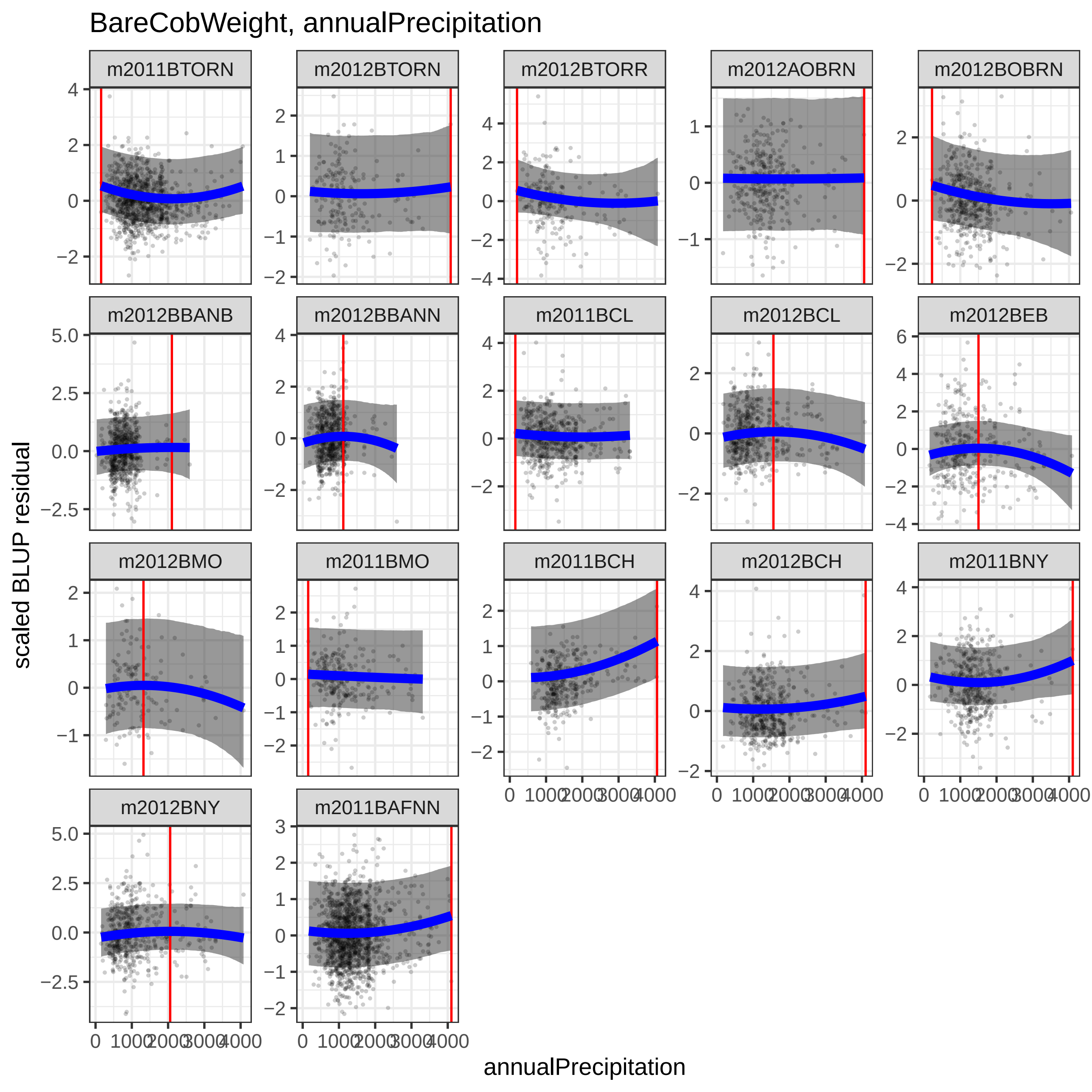

BareCobWeight, annualPrecipitation
m2011BTORN
m2012BTORN
m2012BTORR
m2012AOBRN
m2012BOBRN
m2012BBANB
m2012BBANN
m2011BCL
m2012BCL
m2012BEB
m2012BMO
m2011BMO
m2011BCH
m2012BCH
m2011BNY
m2012BNY
m2011BAFNN
scaled BLUP residual
annualPrecipitation
0 1000 2000 3000 4000

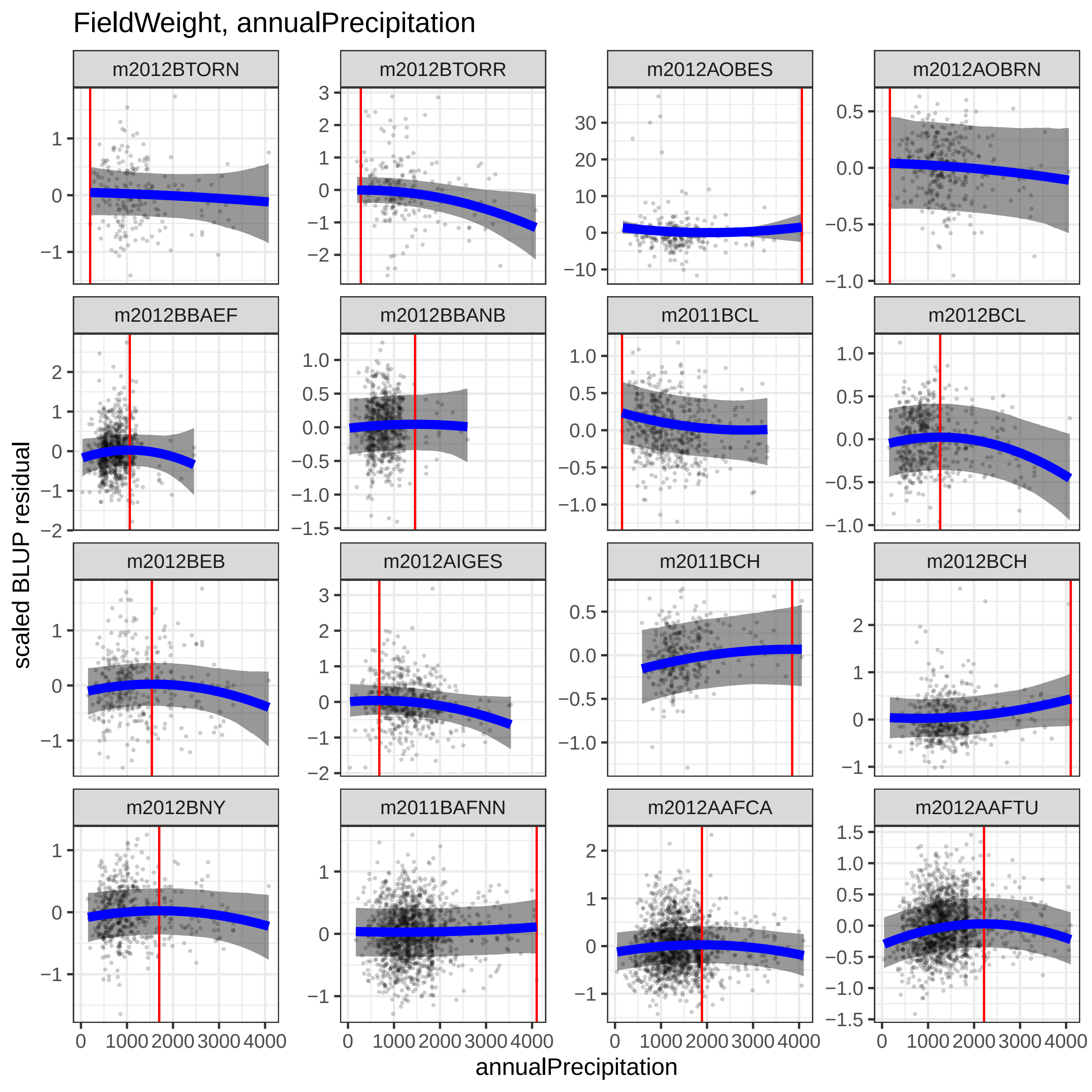

FieldWeight, annualPrecipitation
m2012BTORN
m2012BTORR
m2012AOBES
m2012AOBRN
m2012BBAEF
m2012BBANB
m2011BCL
m2012BCL
m2012BEB
m2012AIGES
m2011BCH
m2012BCH
m2012BNY
m2011BAFNN
m2012AAFCA
m2012AAFTU
scaled BLUP residual
annualPrecipitation
0
1000
2000
3000
4000

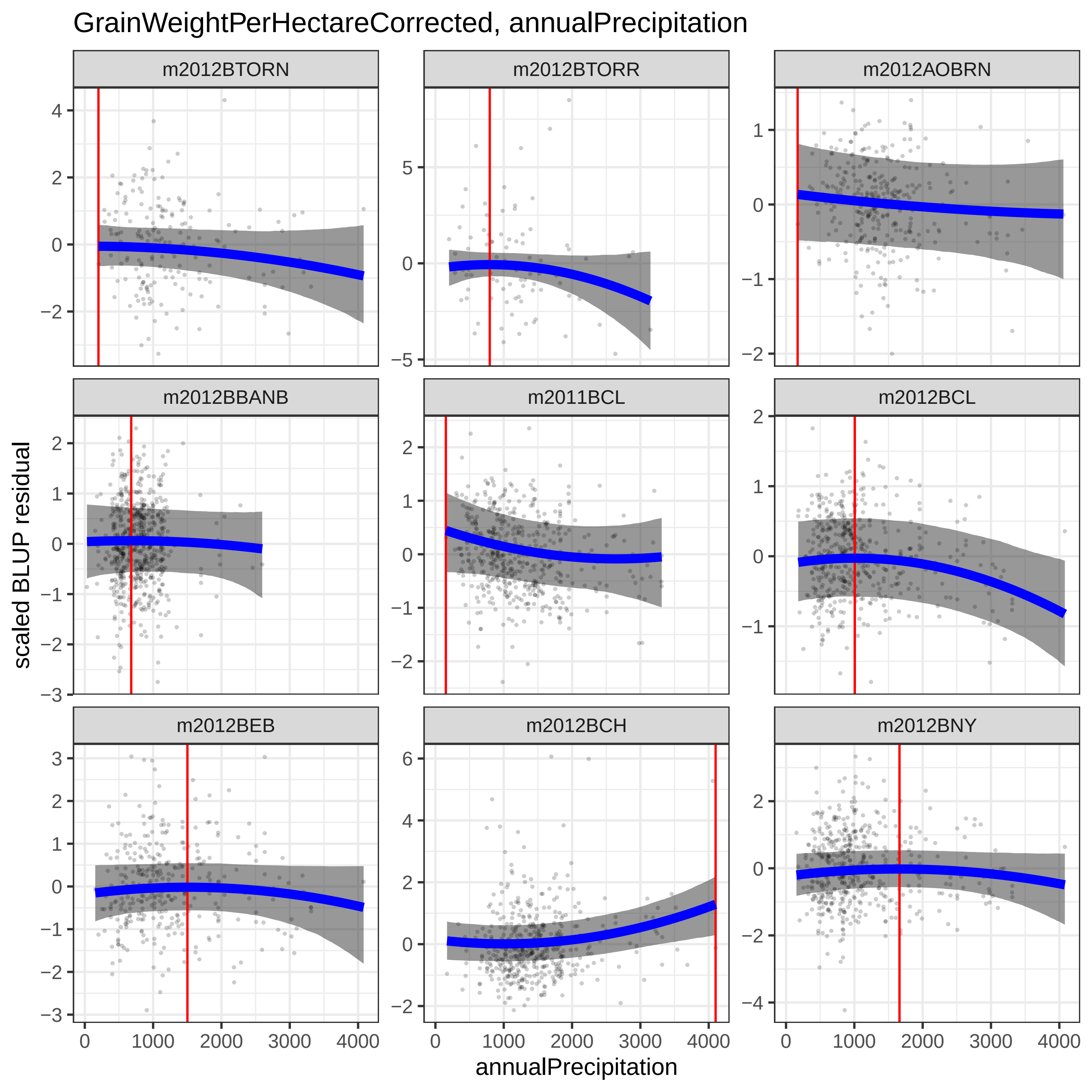

GrainWeightPerHectareCorrected, annualPrecipitation
m2012BTORN
m2012BTORR
m2012AOBRN
m2012BBANB
m2011BCL
m2012BCL
m2012BEB
m2012BCH
m2012BNY
scaled BLUP residual
annualPrecipitation

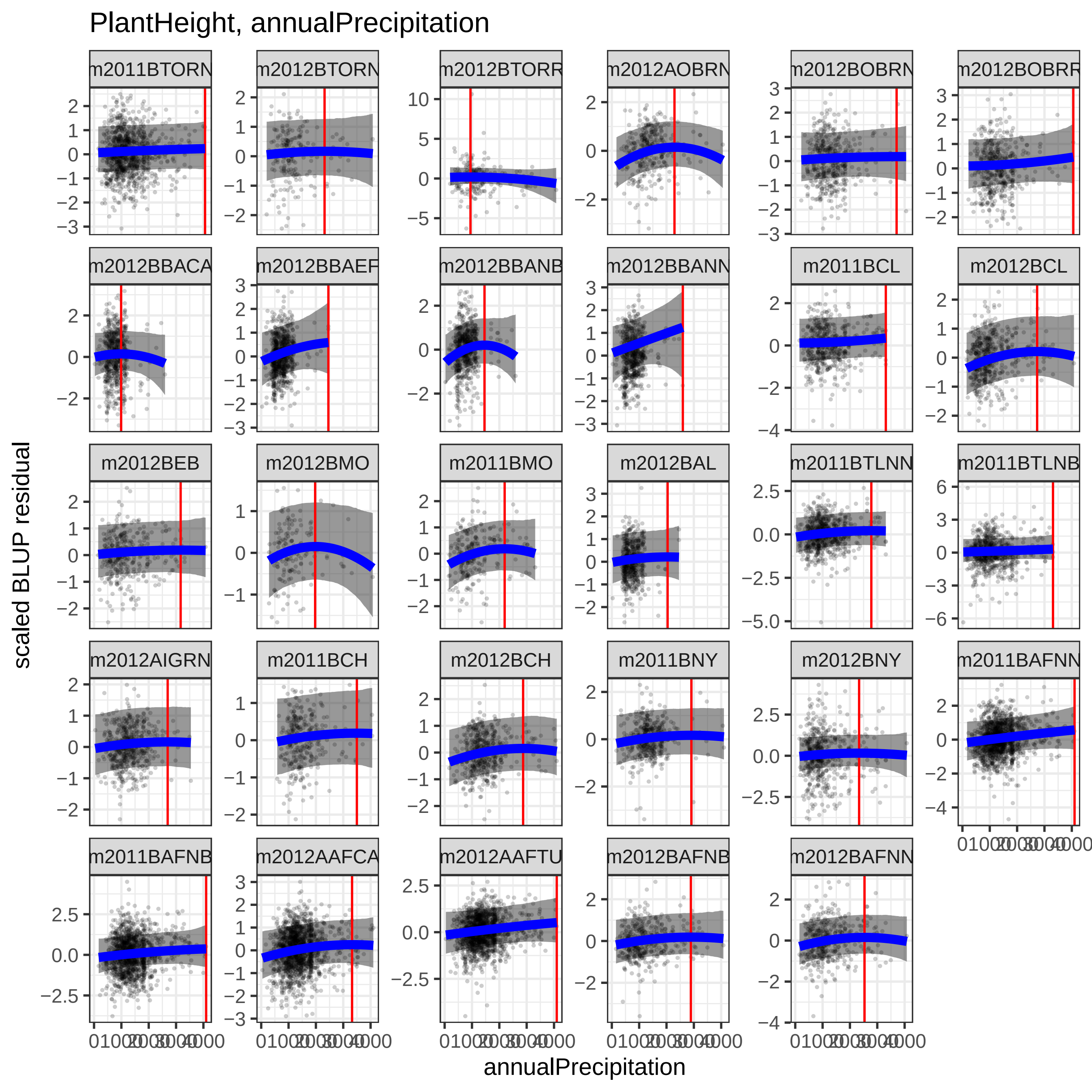
PlantHeight, annualPrecipitation
m2011BTORN
m2012BTORN
m2012BTORR
m2012AOBRN
m2012BOBRN
m2012BOBRR
m2012BBACA
m2012BBAEF
m2012BBANB
m2012BBANN
m2011BCL
m2012BCL
m2012BEB
m2012BMO
m2011BMO
m2012BAL
m2011BTLNN
m2011BTLNB
m2012AIGRN
m2011BCH
m2012BCH
m2011BNY
m2012BNY
m2011BAFNN
m2011BAFNB
m2012AAFCA
m2012AAFTU
m2012BAFNB
m2012BAFNN
scaled BLUP residual
annualPrecipitation

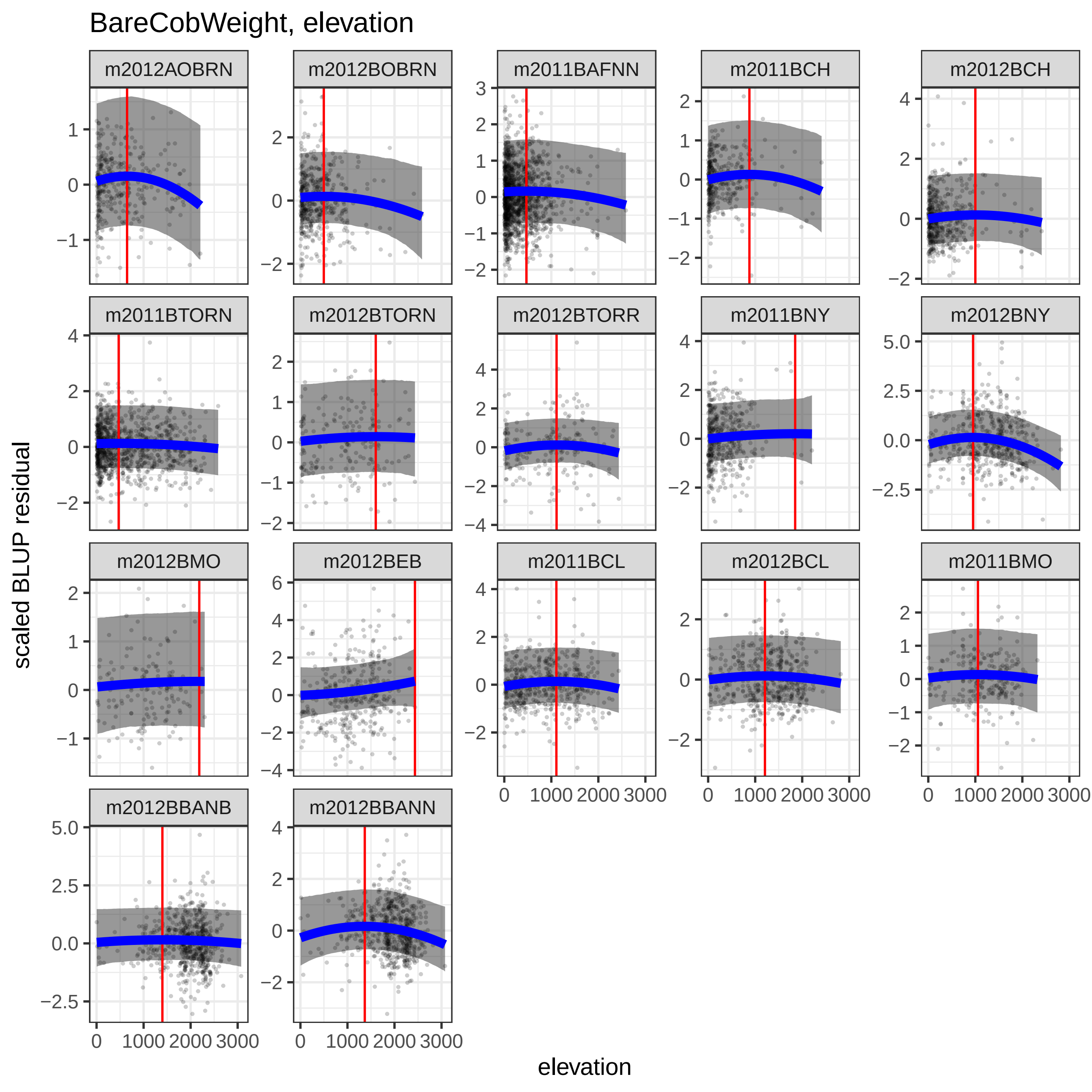
BareCobWeight, elevation
m2012AOBRN
m2012BOBRN
m2011BAFNN
m2011BCH
m2012BCH
m2011BTORN
m2012BTORN
m2012BTORR
m2011BNY
m2012BNY
m2012BMO
m2012BEB
m2011BCL
m2012BCL
m2011BMO
m2012BBANB
m2012BBANN
scaled BLUP residual
elevation
0
1000
2000
3000

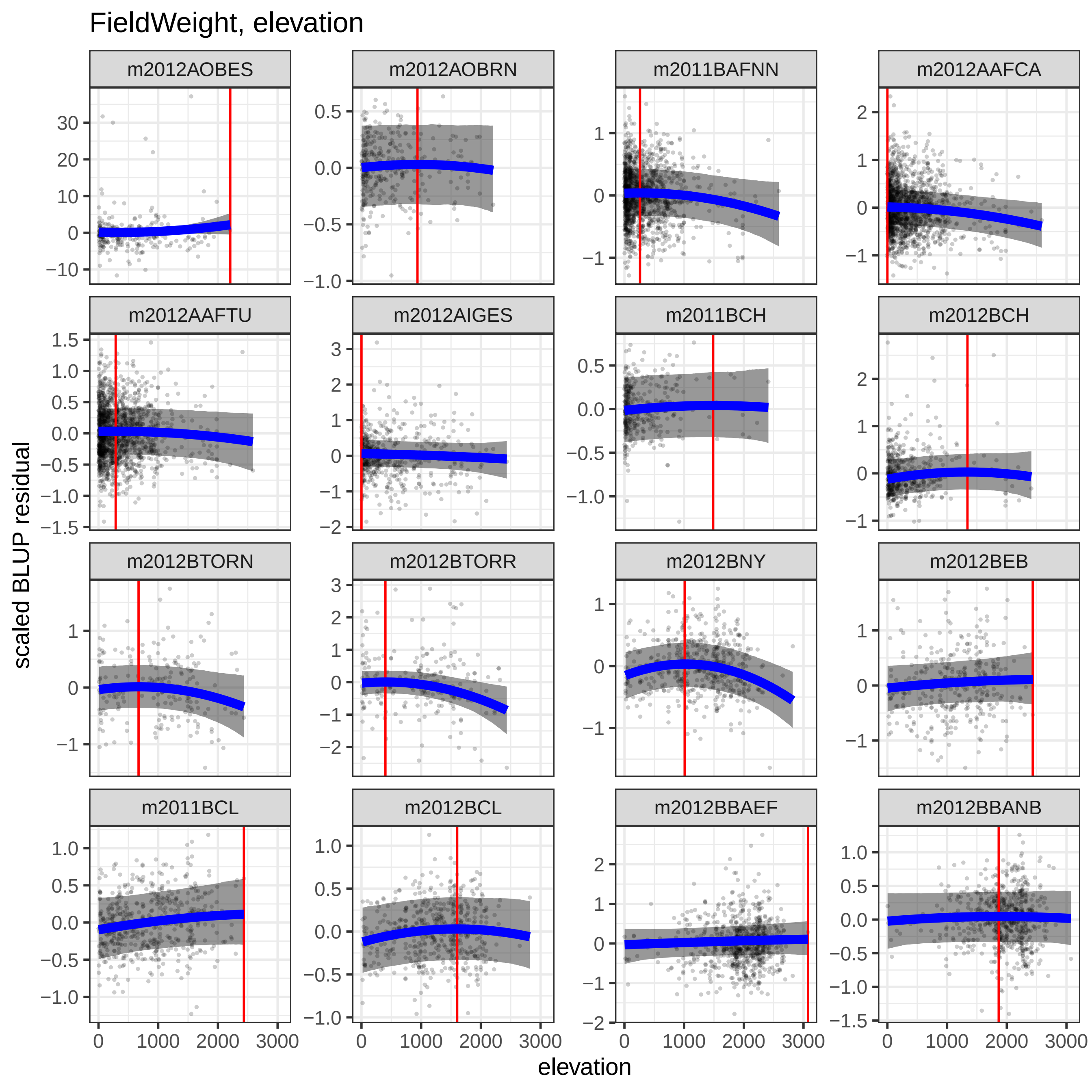
FieldWeight, elevation
m2012AOBES
m2012AOBRN
m2011BAFNN
m2012AAFCA
m2012AAFTU
m2012AIGES
m2011BCH
m2012BCH
m2012BTORN
m2012BTORR
m2012BNY
m2012BEB
m2011BCL
m2012BCL
m2012BBAEF
m2012BBANB
scaled BLUP residual
elevation

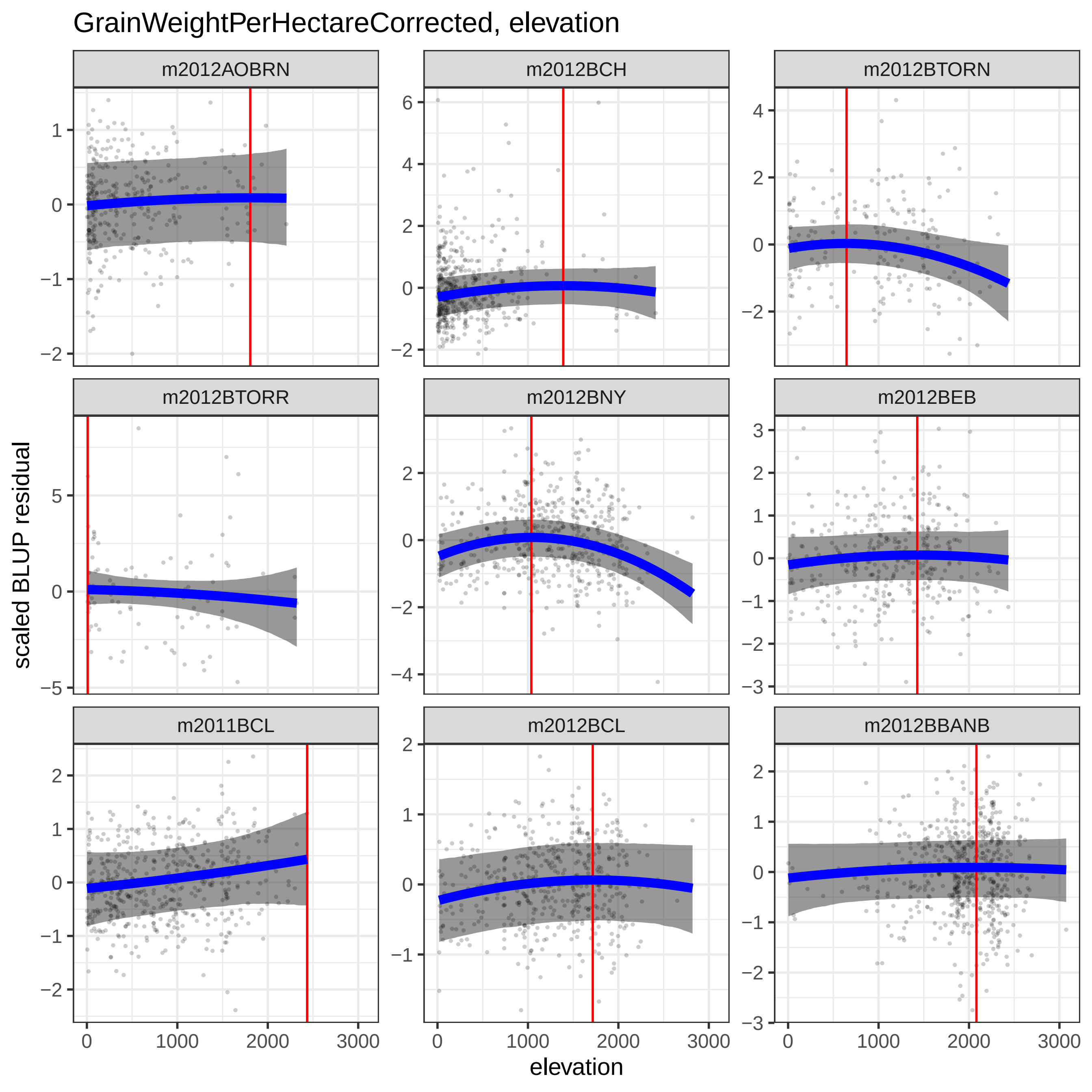
GrainWeightPerHectareCorrected, elevation
m2012AOBRN
m2012BCH
m2012BTORN
m2012BTORR
m2012BNY
m2012BEB
m2011BCL
m2012BCL
m2012BBANB
scaled BLUP residual
elevation
0
1000
2000
3000

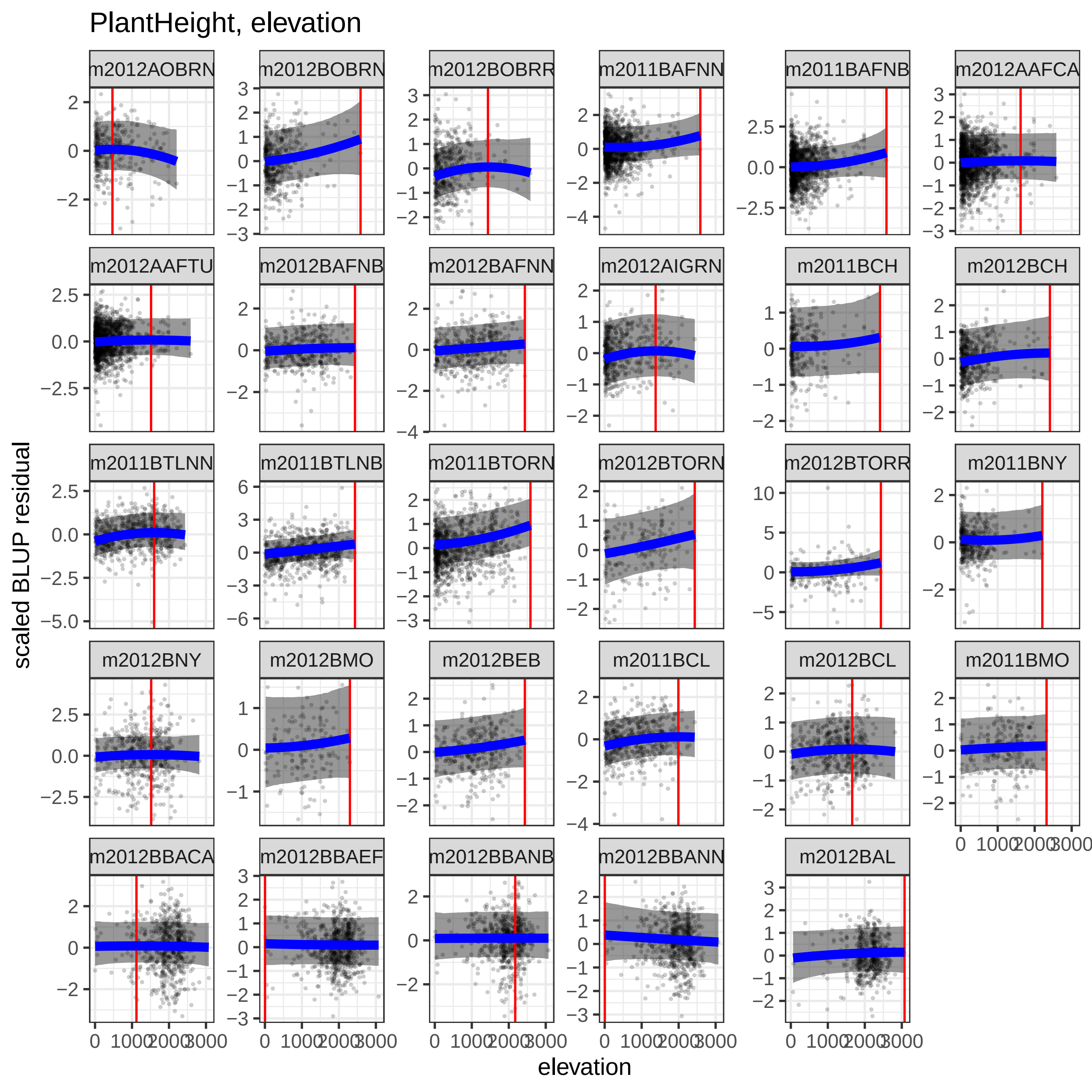
PlantHeight, elevation
m2012AOBRN
m2012BOBRN
m2012BOBRR
m2011BAFNN
m2011BAFNB
m2012AAFCA
m2012AAFTU
m2012BAFNB
m2012BAFNN
m2012AIGRN
m2011BCH
m2012BCH
m2011BTLNN
m2011BTLNB
m2011BTORN
m2012BTORN
m2012BTORR
m2011BNY
m2012BNY
m2012BMO
m2012BEB
m2011BCL
m2012BCL
m2011BMO
m2012BBACA
m2012BBAEF
m2012BBANB
m2012BBANN
m2012BAL
scaled BLUP residual
elevation

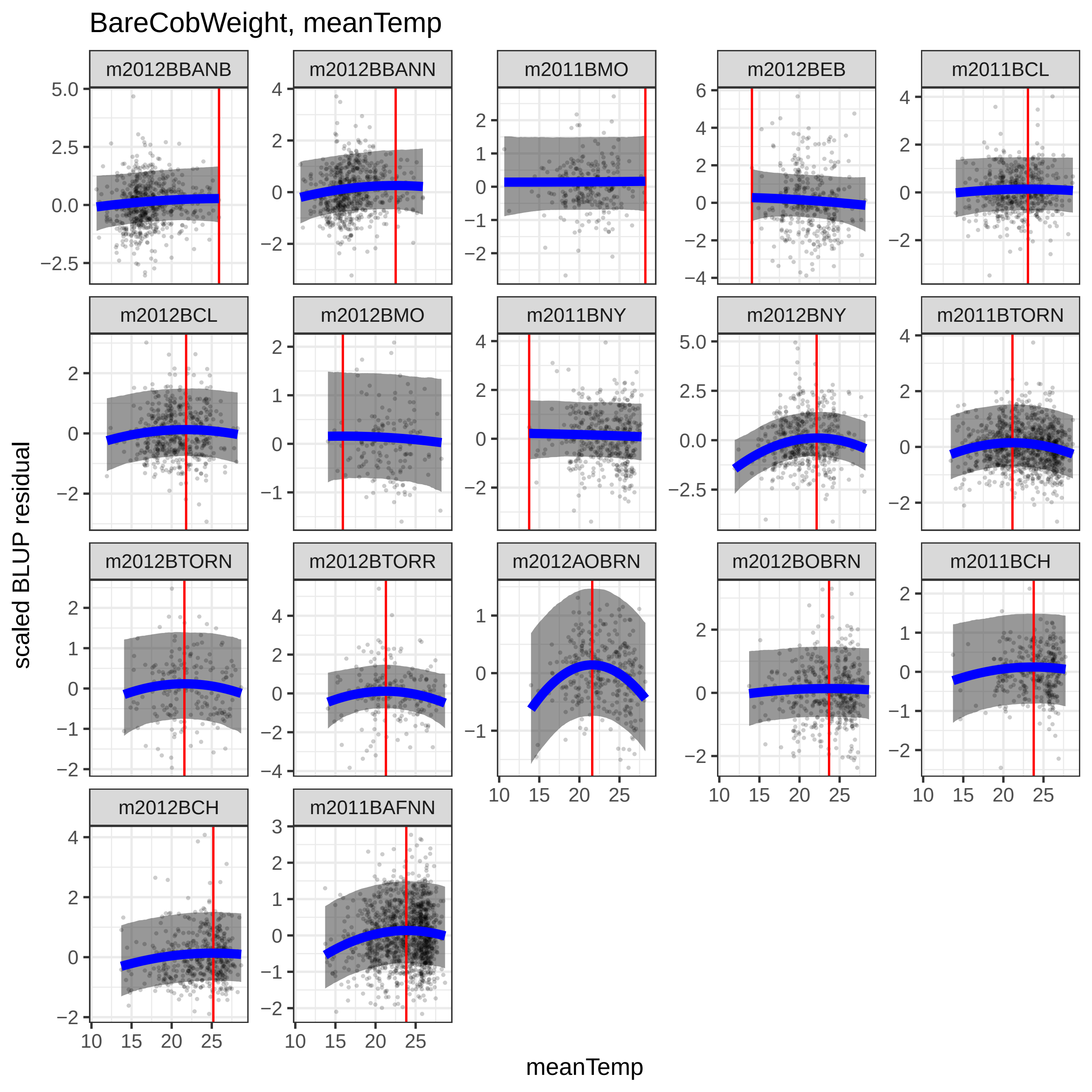
BareCobWeight, meanTemp
m2012BBANB
m2012BBANN
m2011BMO
m2012BEB
m2011BCL
m2012BCL
m2012BMO
m2011BNY
m2012BNY
m2011BTORN
m2012BTORN
m2012BTORR
m2012AOBRN
m2012BOBRN
m2011BCH
m2012BCH
m2011BAFNN
scaled BLUP residual
meanTemp

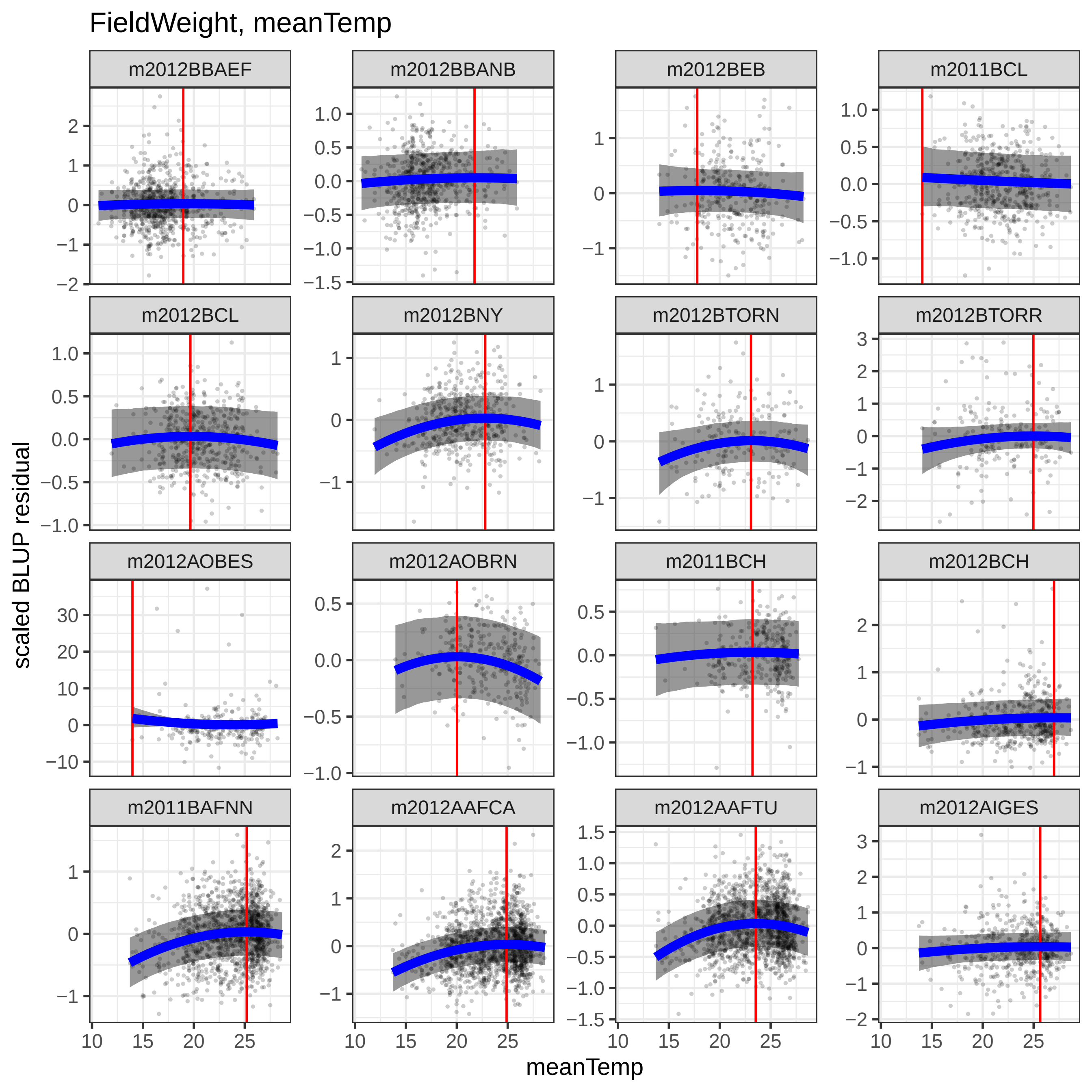

FieldWeight, meanTemp
m2012BBAEF
m2012BBANB
m2012BEB
m2011BCL
m2012BCL
m2012BNY
m2012BTORN
m2012BTORR
m2012AOBES
m2012AOBRN
m2011BCH
m2012BCH
m2011BAFNN
m2012AAFCA
m2012AAFTU
m2012AIGES
scaled BLUP residual
meanTemp

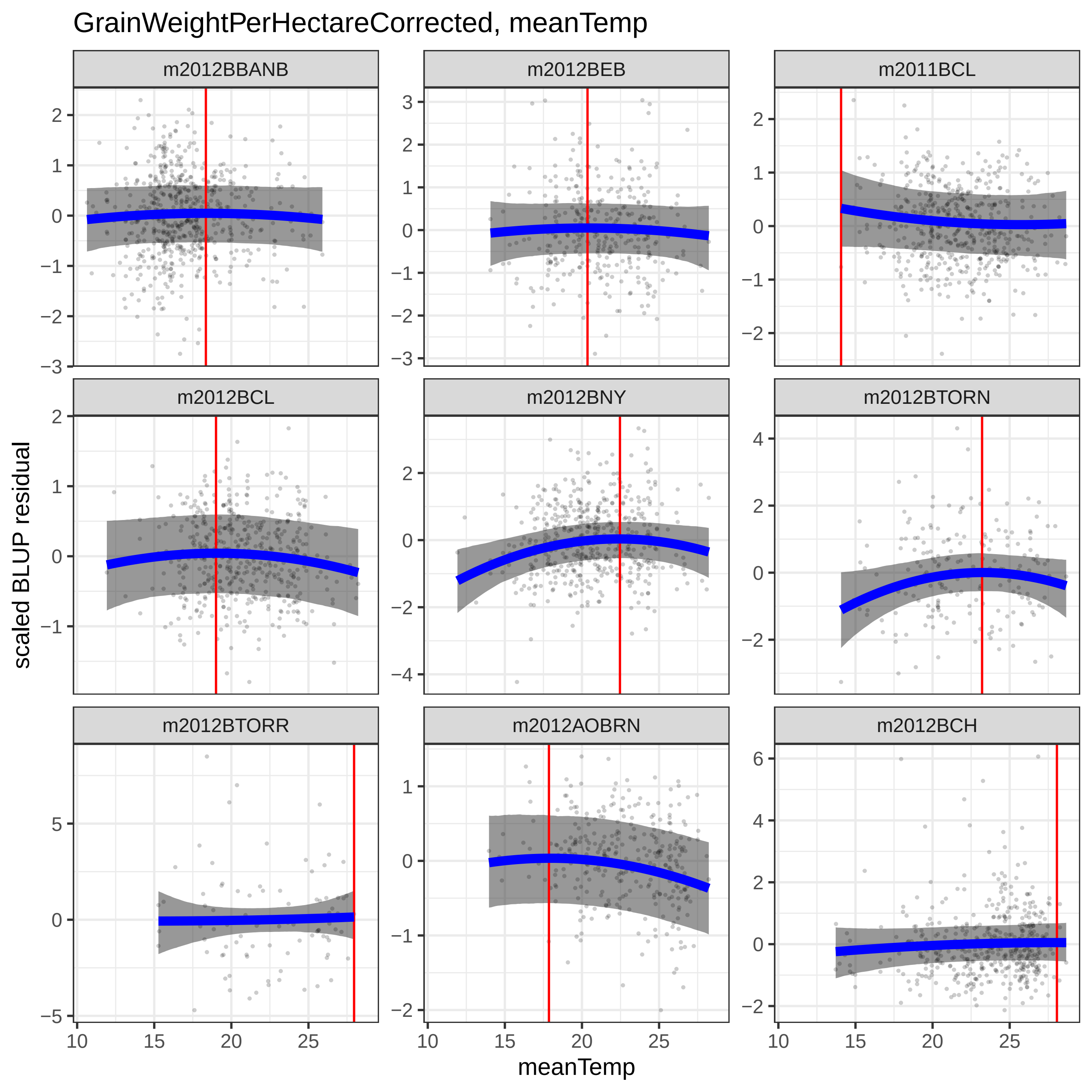

GrainWeightPerHectareCorrected, meanTemp
m2012BBANB
m2012BEB
m2011BCL
m2012BCL
m2012BNY
m2012BTORN
m2012BTORR
m2012AOBRN
m2012BCH
scaled BLUP residual
meanTemp
10
15
20
25

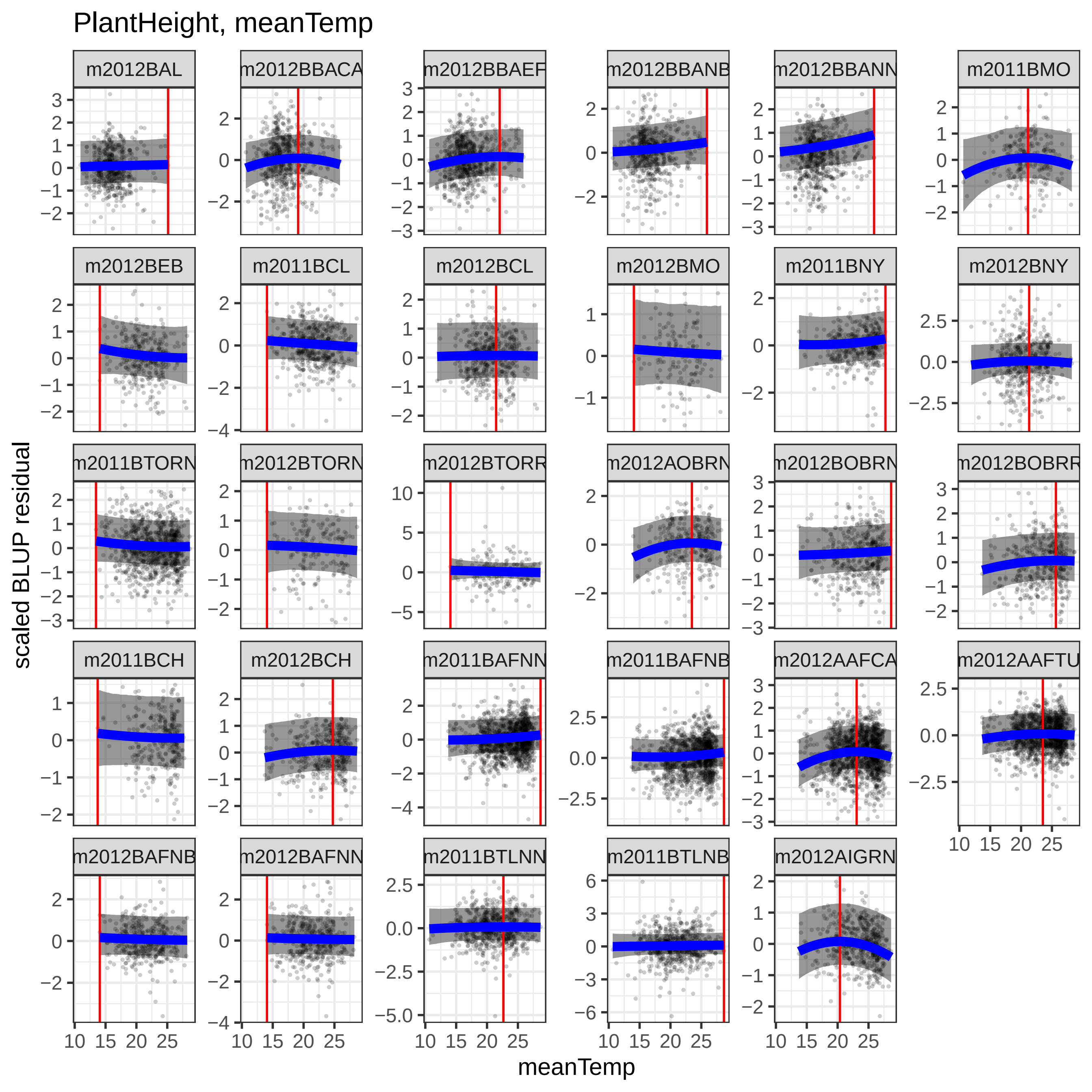

PlantHeight, meanTemp
m2012BAL
m2012BBACA
m2012BBAEF
m2012BBANB
m2012BBANN
m2011BMO
m2012BEB
m2011BCL
m2012BCL
m2012BMO
m2011BNY
m2012BNY
m2011BTORN
m2012BTORN
m2012BTORR
m2012AOBRN
m2012BOBRN
m2012BOBRR
m2011BCH
m2012BCH
m2011BAFNN
m2011BAFNB
m2012AAFCA
m2012AAFTU
m2012BAFNB
m2012BAFNN
m2011BTLNN
m2011BTLNB
m2012AIGRN
scaled BLUP residual
meanTemp
