# Supplementary figures and images for: Environmental data provide marginal benefit for predicting climate adaptation
Source: PLoS Genet. 2025 Jun 9;21(6):e1011714. doi: 10.1371/journal.pgen.1011714 (PMC12173371; doi:10.1371/journal.pgen.1011714)

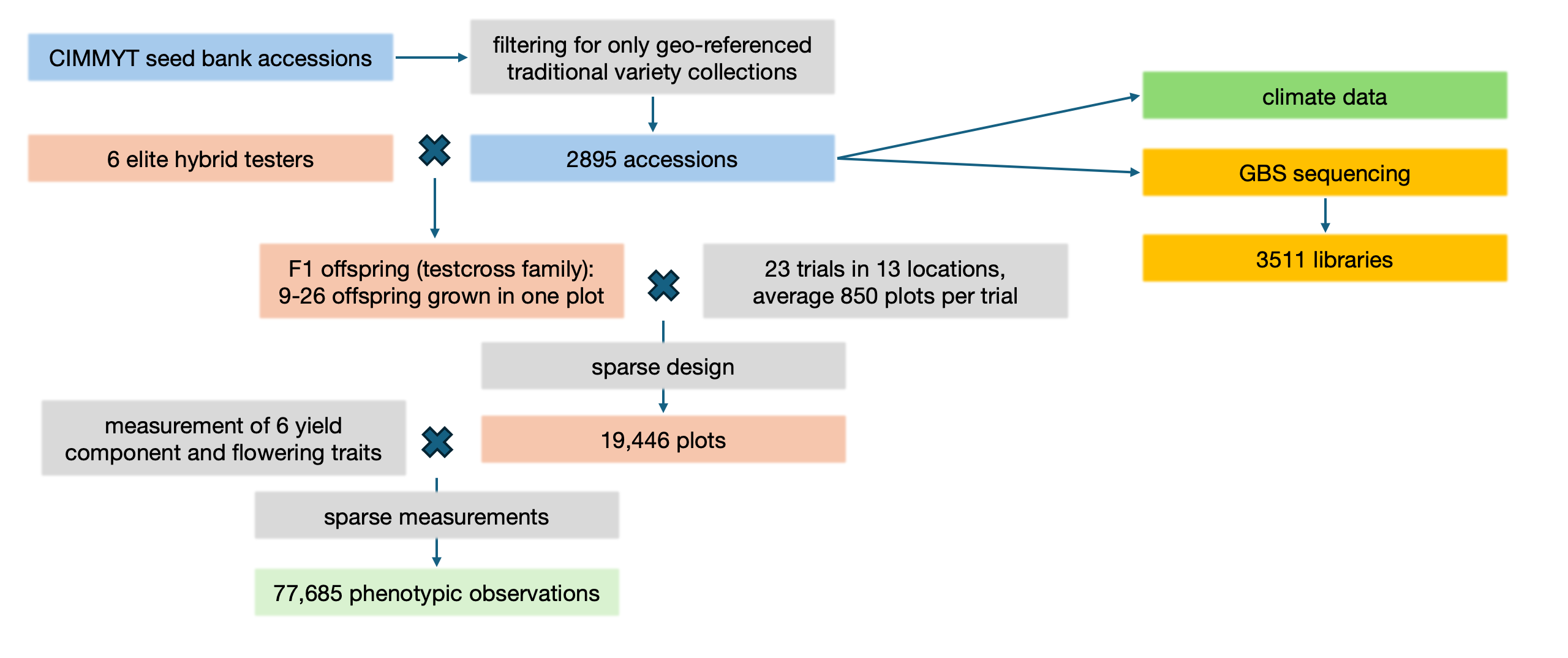

Supplement: S1 Fig — Flowchart visualizing experiment design, including filtering of initial germplasm from CIMMYT, number of testcrosses, plots in locations, and number of observations and genotypes. (TIF) [file pgen.1011714.s008.tif]

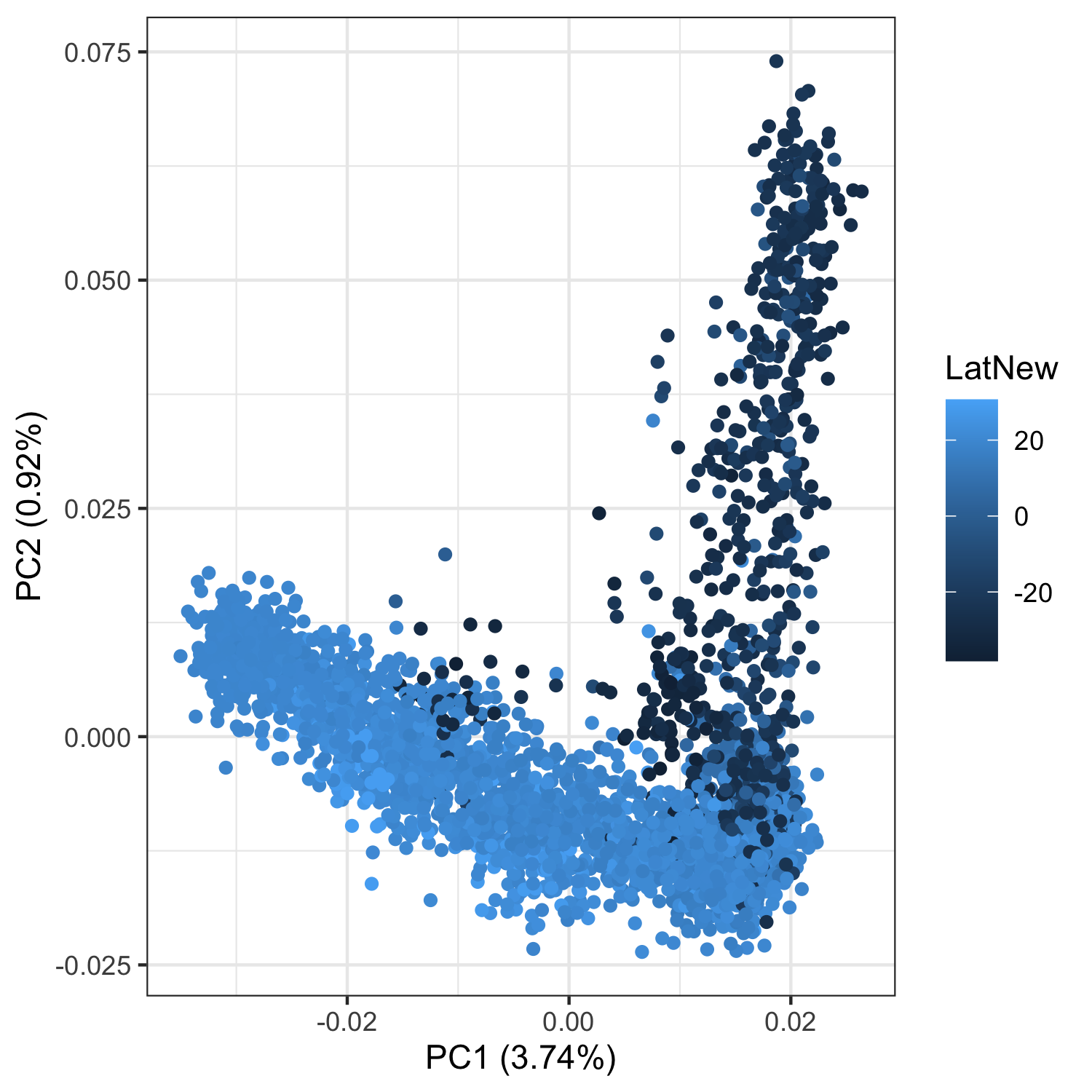

Supplement: S2 Fig — Biplot of PCA1 (representing 3.76% of genetic diversity) vs PCA2 (representing 0.92% of genetic diversity) for all accessions’ genotypes. Points are colored by latitude of collection. (TIF) [file pgen.1011714.s009.tif]

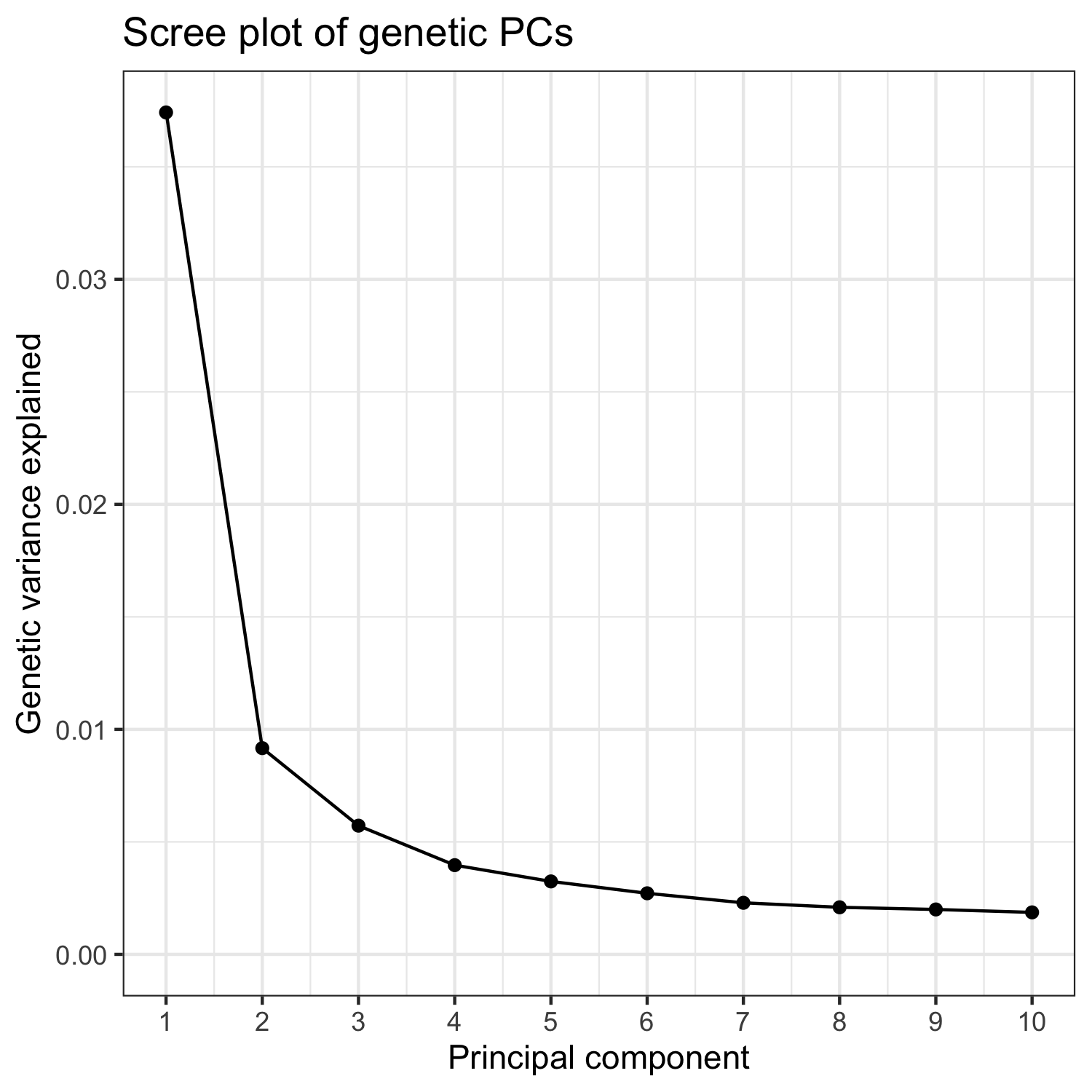

Supplement: S3 Fig — Scree plot of relative contribution of top 10 PCs towards genetic variation in this population. (TIF) [file pgen.1011714.s010.tif]

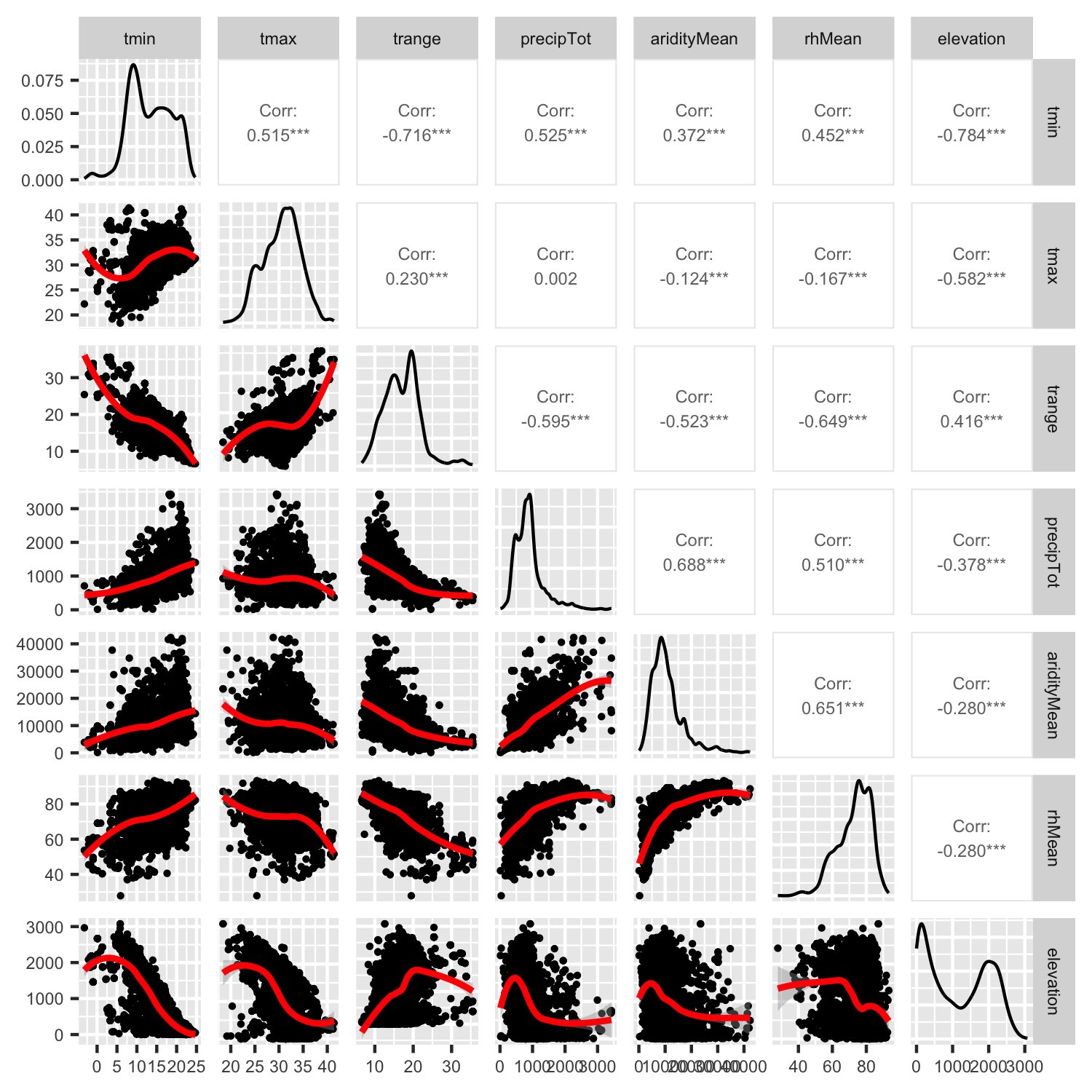

Supplement: S4 Fig — Distributions and correlations between non-INT-transformed growing season environment variables for accessions used in GEA analysis. (TIF) [file pgen.1011714.s011.tif]

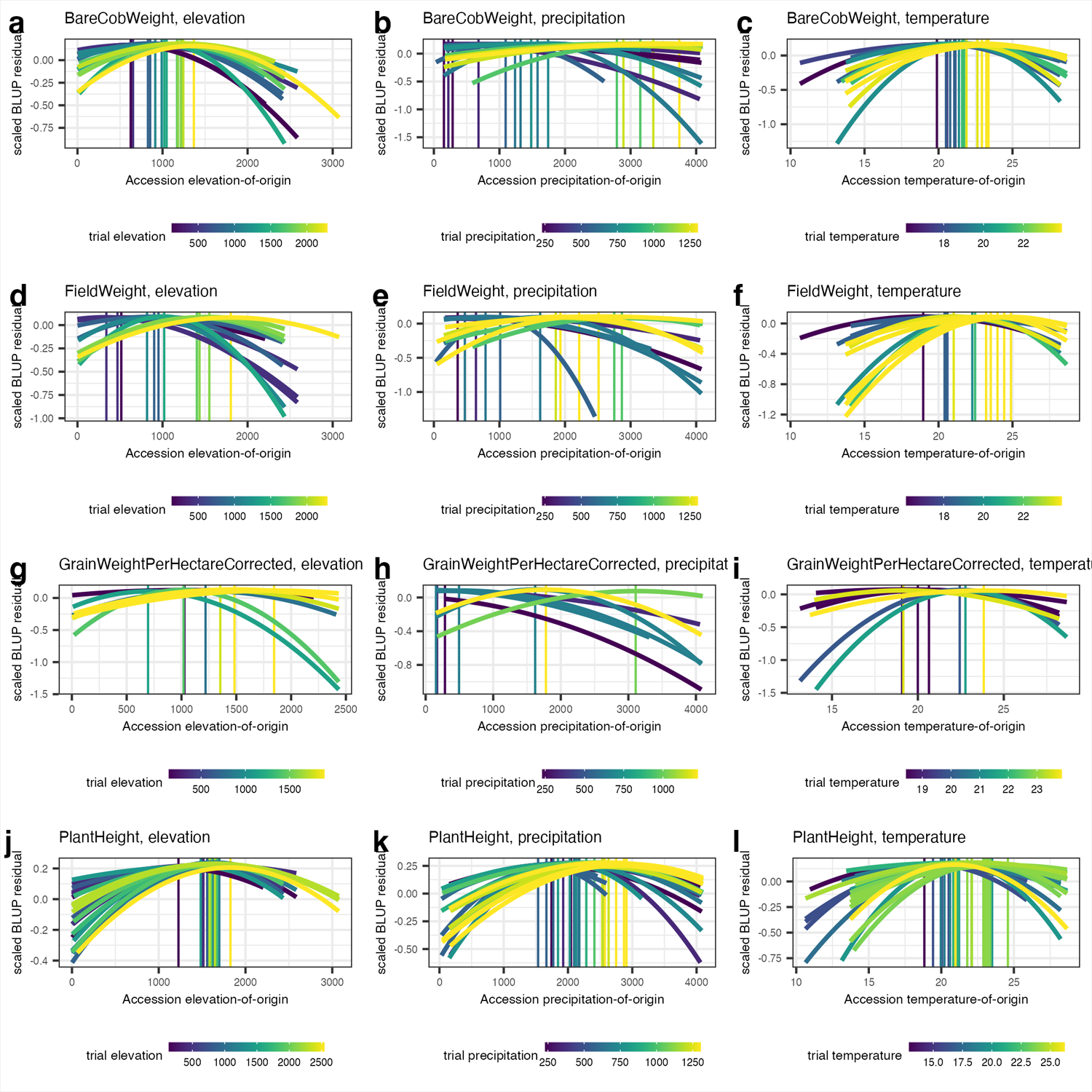

Supplement: S5 Fig — All transfer plot model curves for selected yield component phenotypic trait and environmental variable combinations. Panels a–c measure BLUP residuals for bare cob weight, d–f for field weight, g–i for corrected grain weight, and j–l for plant height. (TIF) [file pgen.1011714.s012.tif]

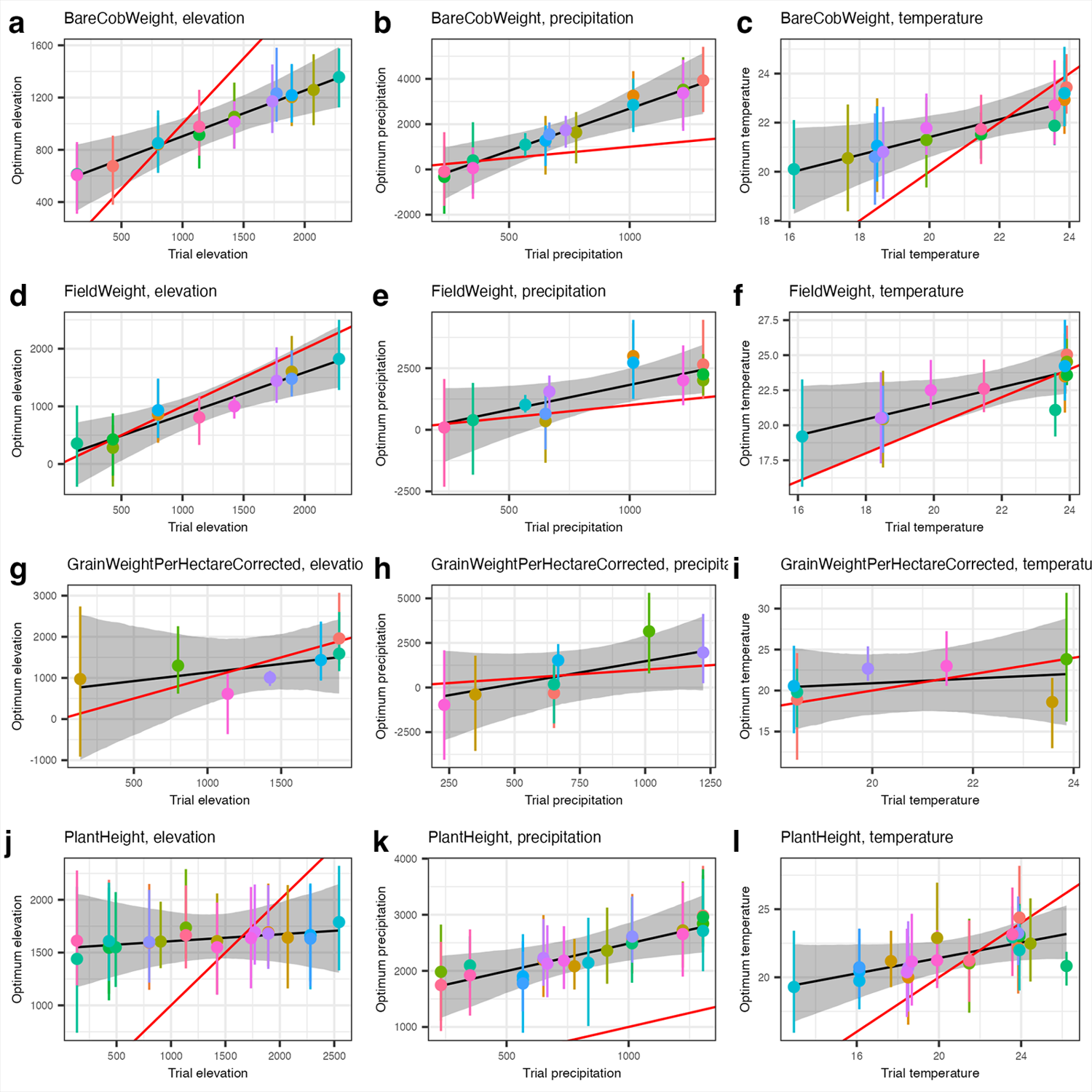

Supplement: S6 Fig — All regression plots of optimal environmental value for a given trial against observed environmental value. Panels a, d, g j measure elevation, b, e, h k measure precipitation, c, f, i, l measure temperature. Individual dots represent trials with measurements for the specific combination. (TIF) [file pgen.1011714.s013.tif]

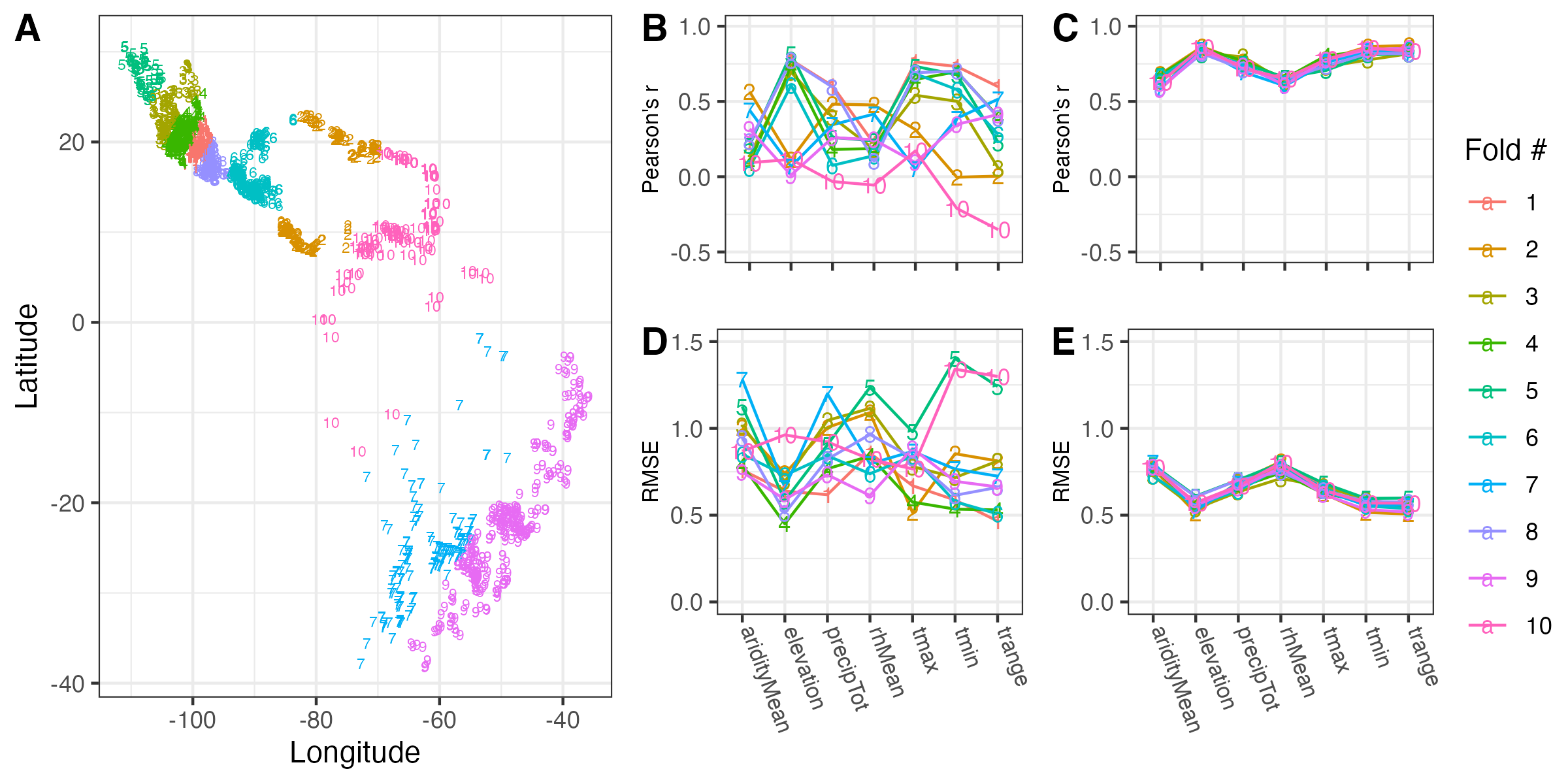

Supplement: S7 Fig — Correlations between genetics and environment are demonstrated through prediction but decrease when considering spatial distance. A) Map of sampled cross-validation folds used in spatial GPoE. (B–E) Pearson’s r correlation and RMSE for spatial (B, D) and random (C, E) sampling for each environmental variable across folds. (TIF) [file pgen.1011714.s014.tif]

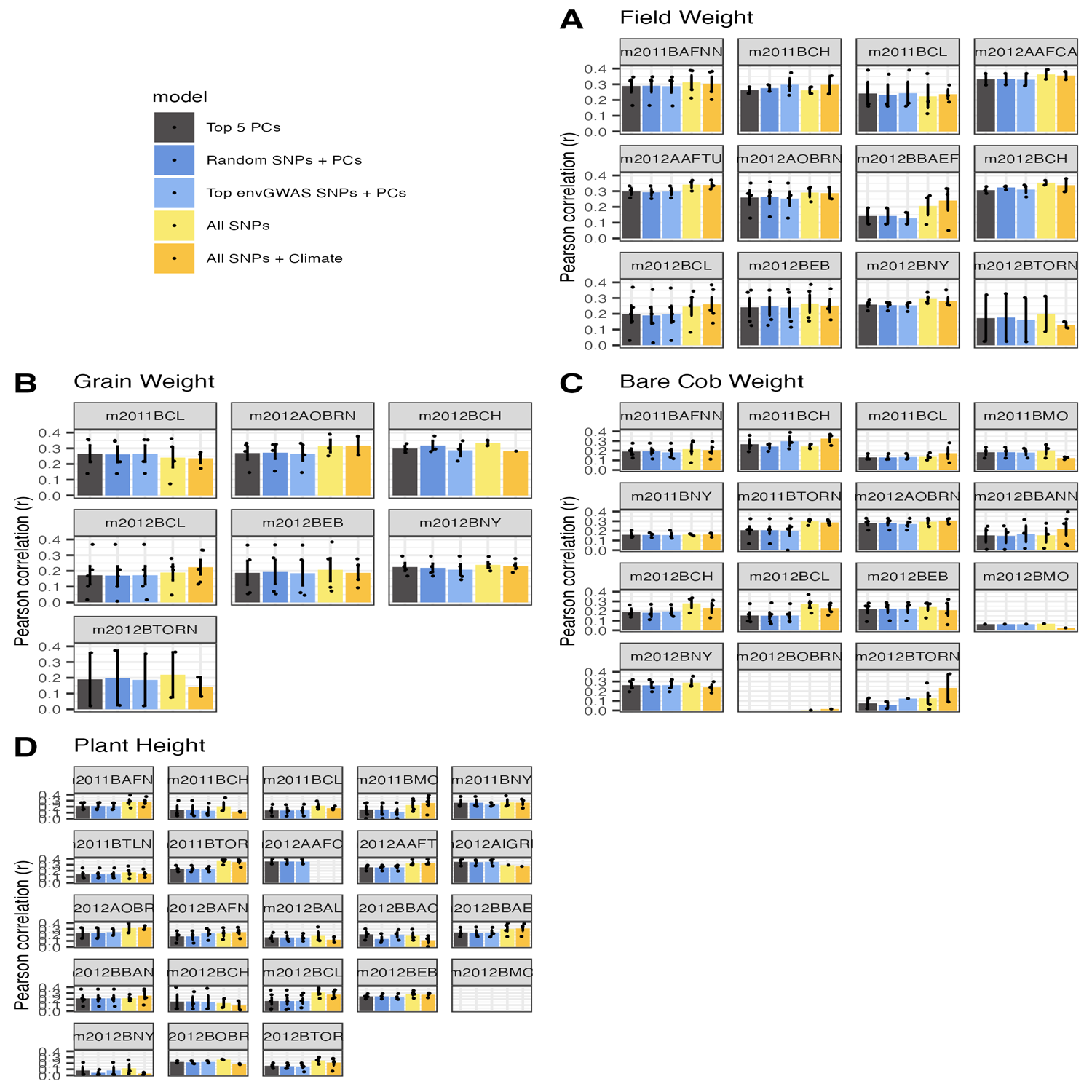

Supplement: S8 Fig — Predictive ability results for field weight, corrected grain weight per hectare, bare cob weight, and plant height, where panels are separated by trial (location and year), measured as Pearson’s r correlation to observed BLUP value. Predictions were made separately for each set of accessions crossed to a given tester for each trial (individual points). Models tested here included top five PCs, random SNPs + PCs, envGWAS SNPs + PCs, all SNPs, and all SNPs + climate data in a linear model. (TIF) [file pgen.1011714.s015.tif]

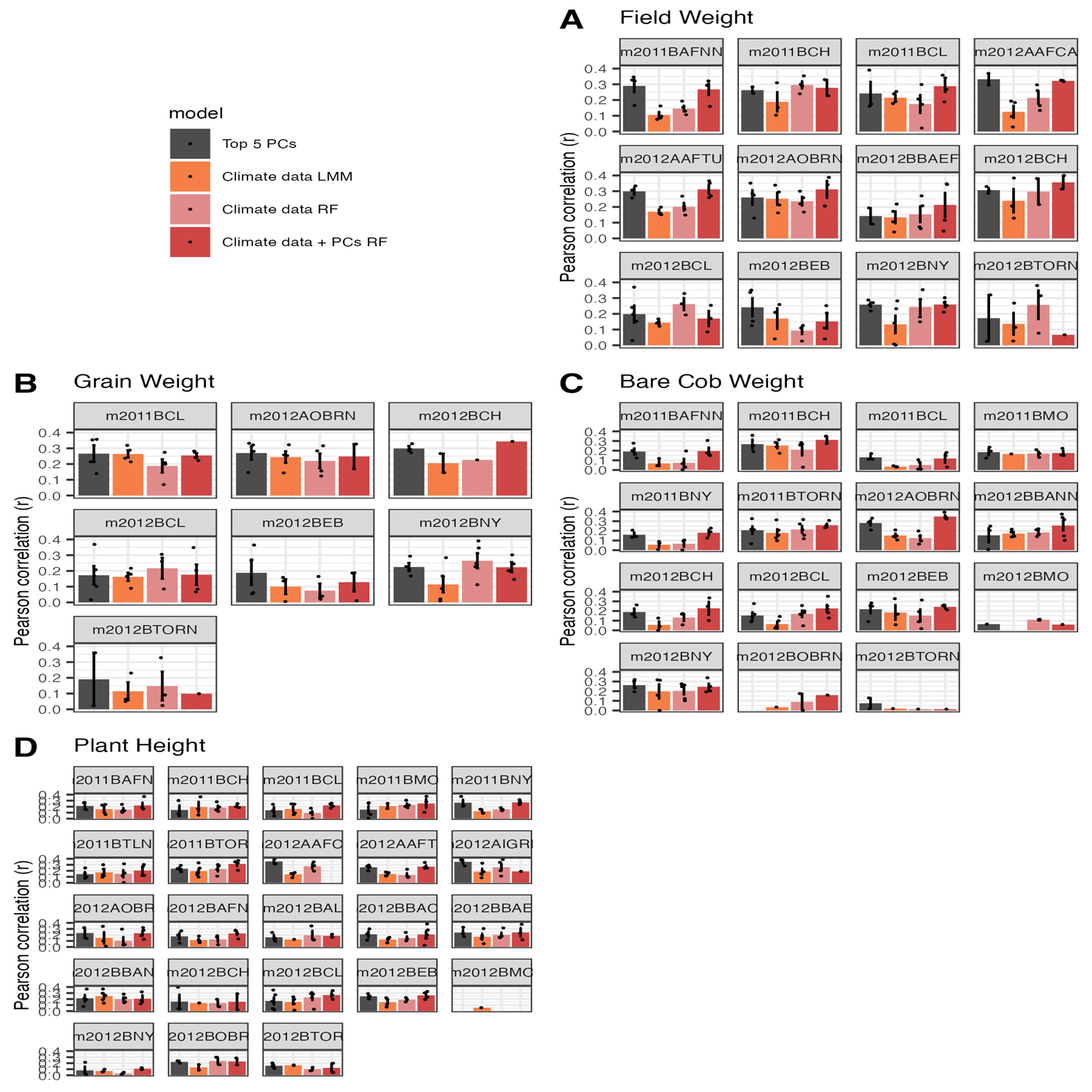

Supplement: S9 Fig — Environmental data model predictive ability results for field weight, corrected grain weight per hectare, bare cob weight, and plant height, where panels are separated by trial (location and year). Models tested here included top five PCs, climate data via linear models, climate data modeled with random forests, and climate data + top five PCs with random forests. (TIF) [file pgen.1011714.s016.tif]

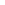

Supplement: S10 Fig — Quantile-quantile plot of multivariate joint envGWAS to assess inflation of p-values. Observed p-value on y-axis plotted against expected p-value on x-axis, with one-to-one line in red. (TIF) [file pgen.1011714.s017.tif]

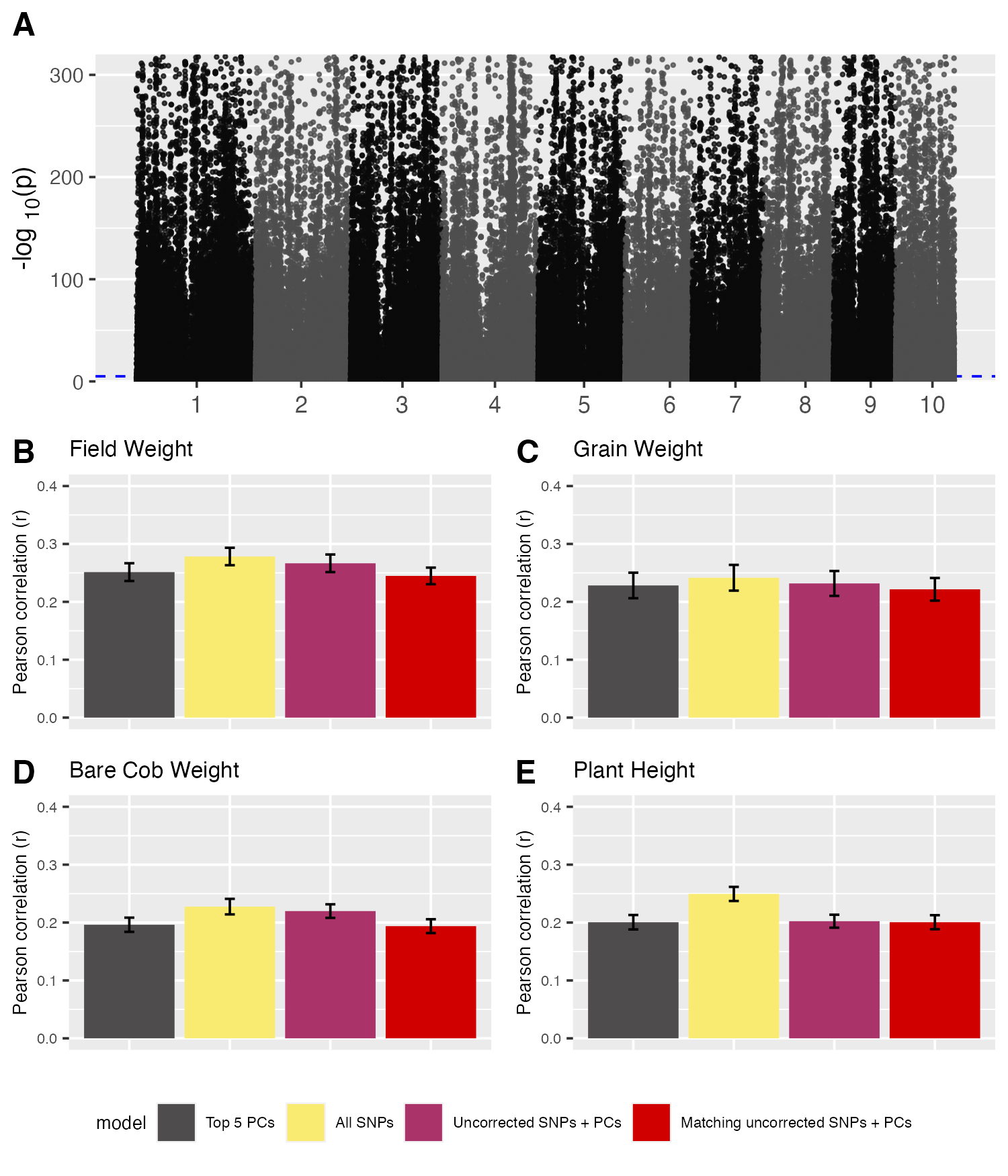

Supplement: S11 Fig — (A) Results from envGWAS without incorporating kinship matrix to control for population structure. (B) Results comparing phenotypic prediction from using top 1000 lead SNPs from unstructured envGWAS + PCs to models including only top 5 PCs (population structure), all SNPs (gBLUP), and a model including random 1000 SNPs of matching allele frequency + PCs. (TIF) [file pgen.1011714.s018.tif]

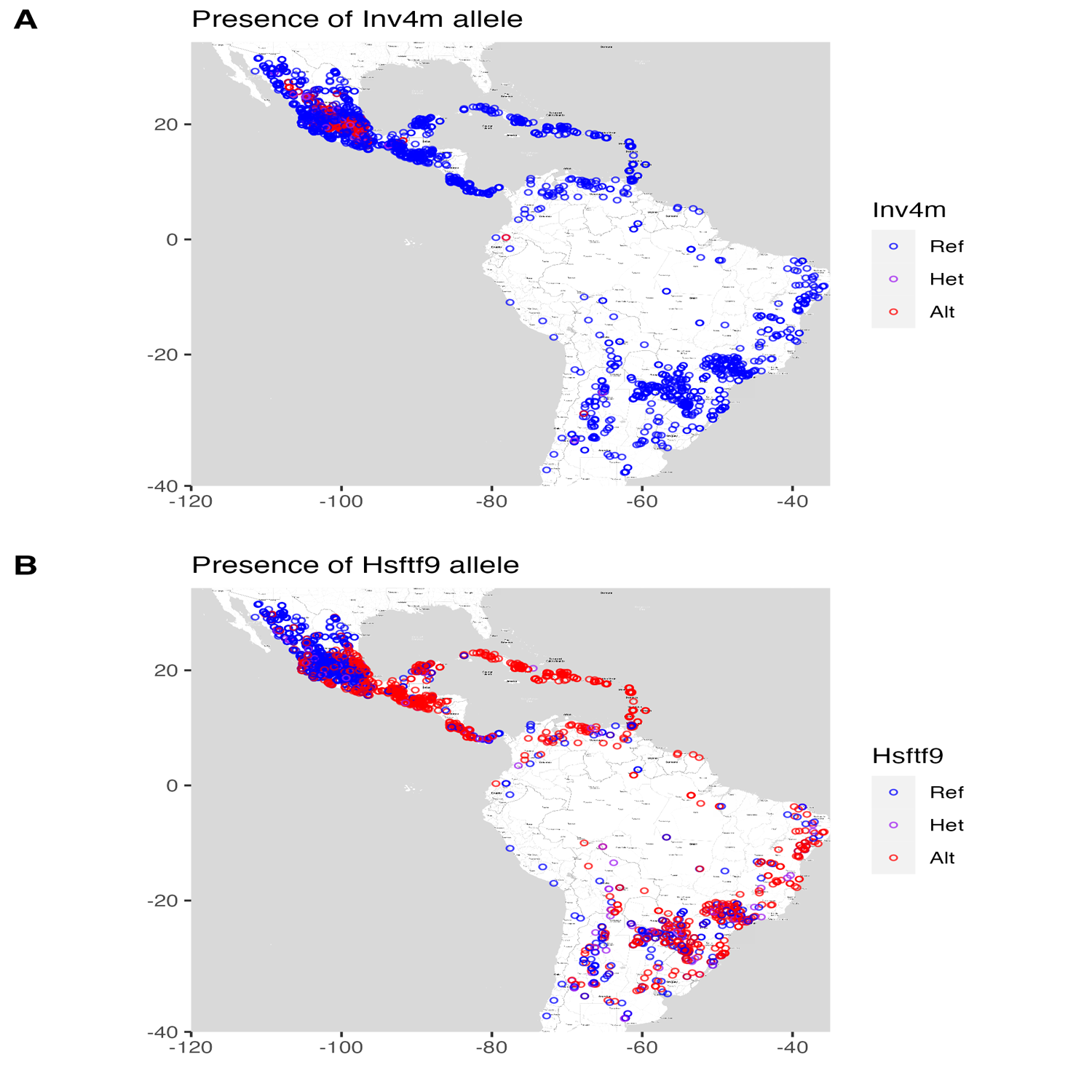

Supplement: S12 Fig — Maps of lead SNP alleles representing (A) the Inv4m inversion and the (B) hsftf9 putative candidate locus. Colors represent number of alternate allele present for given collection accession. Base layer map tiles are from Stamen Design under CC BY 4.0, accessed via the R package ggmap [66]. Data by OpenStreetMap, under ODbL. (TIF) [file pgen.1011714.s019.tif]

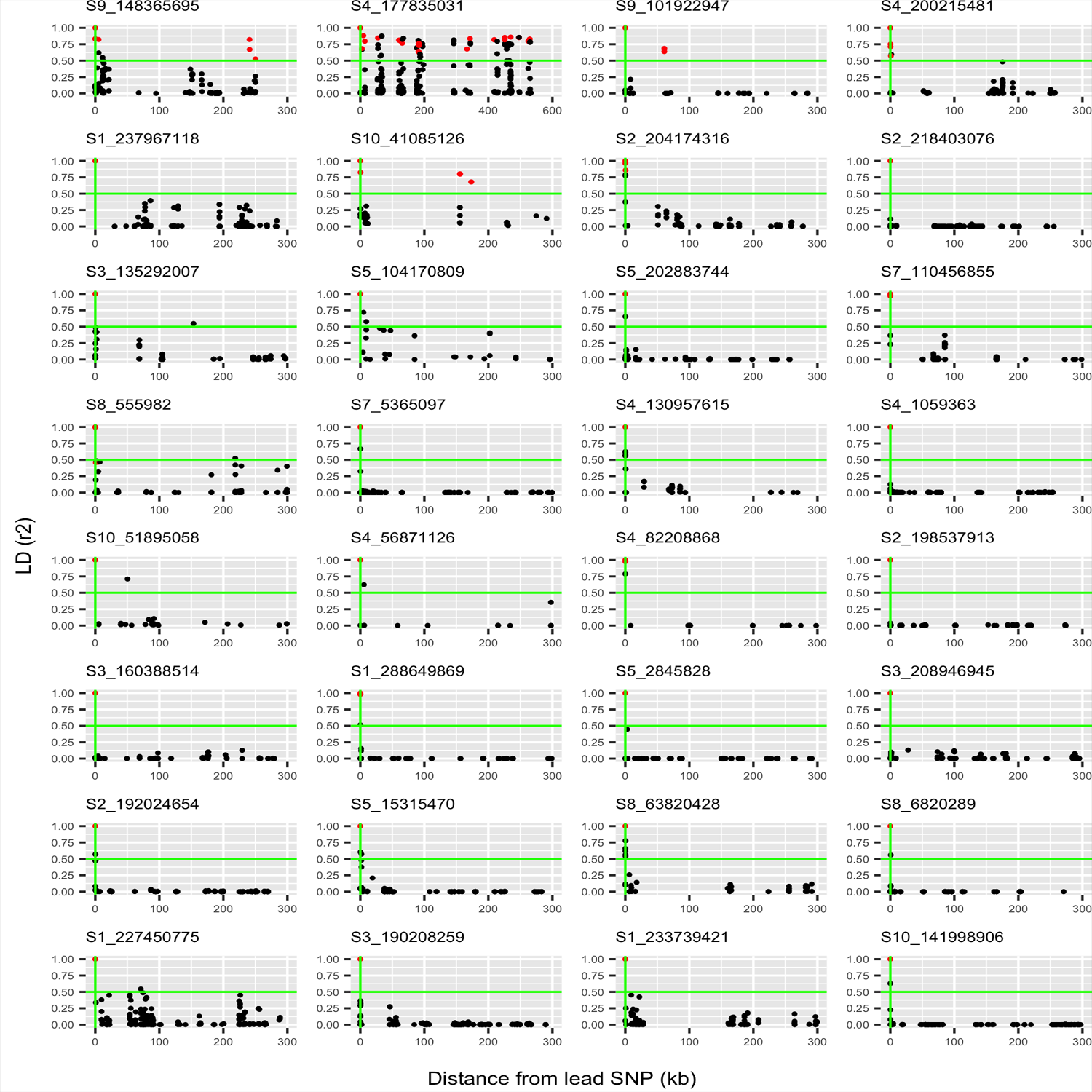

Supplement: S13 Fig — Comparison of LD for all SNPs within ±300kb of top 32 lead SNPs. SNPs found significant in envGWAS are colored in red. For S4_177835031, 600kb was used to describe Inv4m (TIF) [file pgen.1011714.s020.tif]
