# Supplementary material for: Environmental data provide marginal benefit for predicting climate adaptation
Source: PLoS Genet. 2025 Jun 9;21(6):e1011714. doi: 10.1371/journal.pgen.1011714 (PMC12173371; doi:10.1371/journal.pgen.1011714)
Supplement: Attachment — Submitted filename: Response to Reviewers.pdf [file pgen.1011714.s021.pdf]

Response to Reviewers

We thank the reviewers for their feedback on our manuscript.

Reviewer #1: This study evaluated how well genomic and environmental data can be used to predict maize traits, with the goal of evaluating frameworks for identifying traditional varieties into maize breeding programs. The authors are to be commended for pulling together this extensive dataset and for the most part the manuscript was well written. I hope the authors find the following recommends helpful to improve the presentation and rigor of the study.

- The question of how well the environment-of-origin predicts success of a genotype in the field has potential to be very interesting not only to crop researchers, but also to other selective breeding programs (e.g., bovine, aquaculture) and to restoration. The introduction of the manuscript, however, is focused entirely on crops, and the discussion is focused almost entirely on maize. This focus makes the manuscript more suitable for a specialized journal. Majorly revising the intro and discussion to highlight the importance of this topic across multiple different applications would make the manuscript more suitable for a broader PLOS genetics audience.

> We agree with the reviewer that this work contributes useful information to the existing body of work in adapting populations to climate, in both the plant and breeding context as well as in conservation genetics of natural populations. We have altered the title of our manuscript to better address a more generalizable hypothesis regarding how environmental data can be compared to genomic prediction methods - for both breeding and conservation applications. Further, we have added text in both the introduction and discussion covering potential applications in these fields, such as genome-assisted migration of natural populations and previous work looking at genomic prediction from envGWAS-selected loci in tree species. We did not find that our discussion was particularly focused on maize, outside of some caveats regarding previous work with this dataset and related work on similar crop species, but as mentioned, we have added perspectives from other natural populations.

- The main conclusion was that despite a clear signal of local adaptation, neither environment-of-origin nor specific climate-associated SNPs predicted trait yields as well as the top 5 PCs. This seems like a paradox that is not fully addressed in the Discussion. The paradox could potentially be explained by either (1) local adaptation not be as strong as suggested, or (2) these traits being highly polygenic, and thus making it hard to uncover the genetic basis of climate adaptation (e.g. https://www.pnas.org/doi/10.1073/pnas.2220313120). It is also interesting that PCs have this much predictive power despite explaining less than 5% of the genetic variation, and this point should be directly addressed in the discussion.

> We agree with Lotterhos et al. that that these fitness traits in maize are highly polygenic, and may also not be highly correlated with clinal adaptation due to being a cultivated species with high levels of gene flow i.e. trade and migration. We believe that the top 5 PCs explaining ~5% of genetic variation is consistent with having high predictive ability: predictive accuracy = correlation = sqrt(variance explained), so we would estimate 5% of genetic variation explained by PCs would result in a predictive accuracy of 0.22, which is consistent with our results.

- How strong is local adaptation in maize? This is hard to glean from the manuscript for a few reasons. First, the experimental design is intractable from the writing. From the methods, the design sounds highly imbalanced (a fact that the authors were straighforward about), and the use of vague terms such as “trials”, “test cross genotypes” “tester”, “locations”, “plots” and “accessions” make it hard to follow the design. Defining these terms for readers would be helpful. Also, a conceptual figure that encapsulates the experimental design and methods would greatly benefit the manuscript and help it be accessible to a wider audience. While the crossing design might have reduced the signal of adaptation (making estimates of adaptation more conservative), the prior used on the quadratic functions could have made adaptation look stronger than it really is. The curvature of the quadratic function was constrained to be negative through its prior, which is why all the curves on Figure 2 look flat or negative. I would be hesitant to accept this manuscript (1) without a less constrained analysis on the curvature prior and (2) without a figure 2 that shows both outcome variability and statistical uncertainty (currently only the latter is visualized, https://www.pnas.org/doi/10.1073/pnas.2302491120). I suspect that such a figure would be messy, but that would be the point - there is local adaptation, but there is a lot of variability, which is why environment-of-origin does not offer much predictive power.

> The crossing scheme in our experimental design, while a reduction in power as compared to traditional varieties parental generations, is useful for the purpose of elucidating specific loci that may be adaptive and for evaluating breeding values in field trials. We acknowledge that this schema may be confusing and have further defined terms such as “test cross genotypes”, “tester”, and “plots” for a broader PLOS genetics audience. We have also added an additional Supplemental Figure S1 that better visualizes the experimental design.

> Our negative constraint on our transfer distance curves was based on a model in which fitness-related traits must decline when trial environments diverge to an extreme degree from the common garden environments. However, this is predicated on a model that assumes that most trials exhibit local adaptation with a maxima near a predicted optimum. In addition, trials that show a positive curvature may in reality reflect the left- or right-side of a parabola with negative curvature, i.e. we do not have the range in genotype environment-of-origin (e.g. accessions with negative precipitation-of-origin, all accessions are adapted to max temperature below 30 C) for these models to generate a parabola with decreasing fitness in both directions. To help justify this, we included a Supplemental Figure S5 where we modeled local adaptation curves independently for each trial (i.e. without including the trial environmental variable term) and without a negative constraint on the curvature prior. We have also included outcome variability in these plots. We show that there are indeed some trials in which curvature appears flat or positive but that most trials for most trait and environmental clines are negative, suggesting that our combined model with a negative constraint is reasonable.

- Some minor edits would greatly improve the interpretability of the results. For instance, while it’s straighforward to think about the optimum environment for a given accession/genotype, it’s confusing to think about an “optimum environment for a trial”. It’s also confusing to interpret the “slope of the regression of the apex” and why positive B_h indicates local adaptation - it seems

that larger negative values of a_i would be more interpretable. I also found the “spatial CV GPoE” model hard to understand until I read the methods - maybe you mean “leave-one-population-out”? The CV results would also be interesting to a broader audience, as they suggest that predictive values decline rapidly with extrapolation to new populations/environments, and I recommend adding some more on this to the discussion.

> To help streamline readability and interpretation of our results, we have added additional text explaining the definitions of optimum in the context of our environmental adaptation plots. We believe that for our purposes, understanding an accession with an environment-of-origin with a collection environment that best corresponds to a given location’s predicted optimum is of interest for both breeding and conservation, as opposed to finding the environment that an ecotype should be placed in. While more extreme values of a_i would provide one way of interpreting adaptation (stronger signal of decreasing fitness), we use the slope of predicted optima against real trial environment to show that there exist patterns of adaptation along environmental clines. We believe it is of more interest to see that there are wider signals and patterns of adaptation across many trial environments and variables than to compare the strength of adaptation (and for aforementioned reasons, this signal may not be particularly strong in specific trials due to noise in data and may be a poor comparison).

> We have also changed the spatial CV GPoE terminology across the manuscript to better reflect the methodology, and have added additional lines in the discussion explaining potential implications regarding our results.

Overall, this is an extensive analysis that challenges some of the conventional wisdom regarding the predictive power of environment-of-origin. With revisions that make the manuscript accessible to a wider audience, this will make a nice contribution to the literature.

Reviewer #2: The authors have conducted an impressively scaled study of local adaptation in maize and the phenotypic and environmental associations present in the genome. The methods are well considered and generally rigourous, the results are interesting, and the manuscript is clearly written. It is well worth exploring whether the environmental GWAS approach can outperform a "naive" genomic selection approach when applied to a breeding context. I think this is a very strong candidate for publication in PLOS Genetics, but there are a few areas I'd be interested to see incorporated or more clearly described, as discussed below. I think much more interesting biology could be studied in terms of the relevance to understanding evolution, but perhaps that is planned for a future paper -- I can see that might fall outside of the main focus of the present work. I would recommend minor revisions, but would particularly be interested in seeing major comment #2 being included in the final paper.

Major comments:

1. Line 620-622: while I can see that this step combining terms X and Trial:X is simpler, it perhaps obscures very different patterns and causalities -- SNPs that are involved in local adaptation would be expected to show a significant interaction term for a trait like height,

whereas SNPs that were universally beneficial would show a significant main effect. Did you explore whether any interesting signal could be ascertained by studying these separately rather than combining them? Also, when associations with phenotypic traits are discussed on line 225-228, is this for the same Fisher-combined probability of X and Trial:X? Also, I'm assuming that the analysis of the 32 loci in terms of genomic prediction is accounting for the fact that these loci may have had significant main or interaction terms for how well they are able to predict phenotype? I couldn't tell from the methods on lines 624-641 but I think based on it just reimplementing the phenotypic GWAS (line 637) then it is accounting for this (?).

> Prior to the final analysis, we studied the genotype and trial:genotype interaction terms separately to identify G and GxE effects, but found little evidence of separate gene-by-environment effects from the field trials in phenotypic GWAS, and thus chose to combine the terms together. As stated, it would enable discovery of both universally beneficial loci and loci with potentially antagonistic pleiotropy simultaneously . The enrichment for phenotypic traits uses the same Fisher-combined probability of X and Trial:X. For the 32 loci selected from envGWAS, genomic prediction of phenotype is agnostic to whether they contribute to main or interaction terms in the various trials.

2. It would be interesting to know how well environmental GWAS method would have worked for the genomic selection methods if the authors had not performed population structure correction. For the reasons the authors outline on line 360, using population structure correction can reduce false positives but at the expense of incurring false negatives (i.e. missing some true positives that covary strongly with population structure). But if you are comparing a set of associated SNPs to all the SNPs in the genome for the purposes of genomic selection (line 255), it hardly matters if the set of associated SNPs includes some false positives that are not actually causal but just associated. I suspect this wouldn't perform any better, but it would be a more convincing assessment of whether the methodologically more involved environmental GWAS approach can do better than naive genomic selection with lots of SNPs.

> In prior papers looking at envGWAS in this dataset (Romero-Navarro 2019), the authors did not control for population structure, leading to highly inflated p-values due to the sample size of the dataset and high correlation to environmental clines due to spatial auto-correlation. As an example, we ran our envGWAS again in MegaLMM without the inclusion of a kinship matrix K (while maintaining the G and R covariance matrices) and show that high amounts of the genome is tagged by an envGWAS not controlled for population structure, with over 1000 clumped lead SNPs of -log(p-value) greater than 250 tagging locations across the genome. To demonstrate how the > 1000 associated SNPs from this analysis perform in genomic prediction, we show in Supplemental Figure SNNN that the SNPs from uncontrolled envGWAS perform roughly the same as PCs, and that upon adding PCs to that model, perform approximately as well all SNPs. We believe this makes sense as utilizing a large number of SNPs across the genome tagging signals of environmental correlation are likely equivalently tagging population structure due to spatial auto-correlation, and would likewise predict phenotype just as well as full GBS data.

Minor comments:

- Figure 2E: seems a bit strange to show negative precipitation on the y-axis.

While optimum precipitation for a trial cannot be negative, some posterior draws for our model do return negative optimum values for trials at low elevations. The optimum precipitation parameter cannot be easily constrained due to inclusion of modeling of the trial elevation.

- line 587: what are t, b, and n? Please define.

n refers to number of genotypes, t the number of environmental variables, and b the number of genotyped SNPs.

- Line 595: I have no idea what the Cholesky combination does, I can't comment on that technical detail, or how it behaves.

Cholesky decomposition is a common linear algebra procedure:
https://en.wikipedia.org/wiki/Cholesky_decomposition

- Line 318: while large effect alleles may be kept rare under purifying/stabilizing selection, they are expected to be enriched under sustained migration-selection balance. See review by Yeaman (2022; Genetics). Similarly, local adaptation is expected to greatly extend blocks of LD around selected loci due to persistent ongoing hitchhiking that never reaches completion (line 320).

The limited evidence available (Gate 2019, Navarro 2017, Buckler 2009) suggests maize adaptation is likely highly polygenic. Sakamoto et al. 2024 show that if a trait is sufficiently polygenic (the mutation rate to the trait is high), no enrichment of large effect, high frequency alleles is observed. We have added text citing this and arguing we think this applies to maize.
