# Supplementary material for: Environmental data provide marginal benefit for predicting climate adaptation
Source: PLoS Genet. 2025 Jun 9;21(6):e1011714. doi: 10.1371/journal.pgen.1011714 (PMC12173371; doi:10.1371/journal.pgen.1011714)
Supplement: Attachment — Submitted filename: Response to Reviewers 4-25-25.pdf [file pgen.1011714.s022.pdf]

Response to Reviewers 4/25/25

We thank the reviewers again for their feedback on the manuscript.

Reviewer #1: The authors have done a satisfactory job addressing the reviewer comments. The dataset and analyses are impressive, and this paper will inform important debates about the utility of envrGWAS SNPs in management.

As a minor comment, the models and results presented on lines 140-155 are still confusing (as well as the relevance of "positive slope parameter" and BH = 1).

> We have attempted to better describe the results and methodology in lines 140-155 as suggested so as to make it more straightforward.

It would be helpful in Figure 2A and B to show the trial elevation in addition to the optimum elevation.

> We have added the actual trial elevation to Fig 2A-C for comparison to the optimum, as suggested.

Reviewer #2: The authors have responded appropriately to all my suggestions, and I appreciate the addition of the new analysis. I have no further concerns about the manuscript and think this is a good candidate for publication.

I checked the data availability statement and the link for the github repo was broken, and the github author's page did not appear to have a corresponding repo. I think that open data is critical for science, and would suggest the authors publish an archive on dryad or similar repository including all of the intermediate data used to generate each figure, along with documentation and metadata required to interpret them. This is a bit of work, but is an important step, beyond the raw sequence data/climate data.

> Upon review of the github repo link in the Data Availability, we found that the link provided in this round of submission should have been correct (https:/github.com/liforrest6/SeeD). Though the first submission included an incorrect author link (liforrest/SeeD), the second submission should have had the correct link both on the editorial submission portal and in the manuscript itself. If this is not the case, we are happy to help fix it, but we were unsure where this confusion occurred in submission.

> We appreciate and agree with the second reviewer's comments on open data availability and reproducibility. However, CIMMYT data includes specific use restrictions

(https://data.cimmyt.org/dataset.xhtml?persistentId=hdl:11529/10548233&version=1.2&selectTab=termsTab) and the raw data can be accessed through their Dataverse repository with permission. In addition, we have uploaded non-identifiable intermediate data that complies with CIMMYT's restrictions for our results and figures onto Dryad: http://datadryad.org/share/DiPTw1nlyNiPmodhvBZcaVeidFmIUTtuQsSflm0rpm8

Again, we thank the reviewers for their invaluable insight and advice.

On behalf of the authors,

Forrest Li
